# Pre-Harvest Aflatoxin Contamination in Crops and Climate Change Factors: A European Overview

**DOI:** 10.3390/toxins17070344

**Published:** 2025-07-08

**Authors:** Ainhoa Bereziartua, Anke Huss, Jannigje G. Kers, Lidwien A. M. Smit, Roel Vermeulen, Daniel Martins Figueiredo

**Affiliations:** 1 Department of Preventive Medicine and Public Health, University of the Basque Country (UPV/EHU), 48940 Leioa, Basque Country, Spain; ainhoa.bereziartua@ehu.eus; 2 Biogipuzkoa Health Research Institute, 20014 San Sebastián, Basque Country, Spain; 3 Institute for Risk Assessment Sciences (IRAS), Utrecht University, Postbus 80125, 3508 TC Utrecht, The Netherlands; a.huss@uu.nl (A.H.); j.g.kers@uu.nl (J.G.K.); l.a.smit@uu.nl (L.A.M.S.); r.c.h.vermeulen@uu.nl (R.V.)

**Keywords:** aflatoxin, pre-harvest contamination, climate change, environmental factors, food safety, data standardisation, Europe

## Abstract

Aflatoxin (AF) contamination of crops during the pre-harvest period is a significant global concern for food and feed safety (FFS). In Europe, climate change presents a growing threat to agricultural products by increasing the risk of AF contamination. This umbrella review evaluates the scope and quality of pre-harvest data on climate-related AF contamination in Europe, addressing key questions: What insights do researchers provide on the relationship between climate change and pre-harvest AF contamination, and what data are lacking? Which crops are the focus of current research, and where in Europe are these studies concentrated? How is the data presented, and is it standardized? We conducted an umbrella literature review, extracting relevant studies from PubMed and Scopus up to 14 October 2024. Our findings indicate that rising temperatures, droughts, and shifting rainfall patterns increasingly favor the growth of aflatoxigenic fungi and pre-harvest AF contamination in European crops, posing risks to FFS and agricultural stability. However, inconsistencies in data collection and reporting limit cross-regional comparisons and hinder the development of effective mitigation strategies. Standardizing methodologies and improving data accessibility will enhance predictive modeling, strengthen risk assessments, and support targeted adaptation efforts, providing actionable insights for policymakers and agricultural stakeholders.

## 1. Introduction

Mycotoxins can contaminate up to 80% of all crops, and approximately 20% of global crops exceed European Union (EU) legal food safety limits [1]. Despite advancements in agronomic practices and food safety measures, mycotoxin contamination remains a major concern for food and feed safety (FFS) worldwide. In Europe, certain mycotoxins are prioritized in food safety regulations due to their toxicity and prevalence in agricultural products. Regulated mycotoxins include aflatoxins (AFs), fumonisins (FUMs), zearalenone (ZEA), ochratoxin A (OTA), patulin, trichothecenes (e.g., T2, HT-2, deoxynivalenol [DON]), ergot alkaloids (EAs), and citrinin (CIT). Other mycotoxins, such as cyclopiazonic acid (CPA), enniatins (ENNs), beauvericin (BEA), sterigmatocystin, phomopsins, and alternaria toxins, remain unregulated, and their toxicological profiles are still under evaluation. Although their full effects on human and animal health are not yet understood, concerns persist regarding their potential impact [2,3,4,5]. AF contamination is of particular concern due to its significant implications for public health. AFs are among the most toxic naturally occurring compounds, attracting considerable attention due to their high toxicity and frequent occurrence—for instance, in milk intended for human consumption [6]. Classified as genotoxic, carcinogenic (Group 1 by the International Agency for Research on Cancer), and immunosuppressive, AFs are associated with both acute and chronic toxicity [7]. Chronic exposure has been linked to liver cancer, developmental impairments in children, and increased vulnerability to infections. Severe acute exposure can cause rapid liver damage, gastrointestinal distress, multi-organ failure, and even death [7,8,9,10].

While Europe benefits from advanced agricultural and storage practices that help reduce post-harvest risks, climate change and shifts in farming methods—such as minimal tillage and reduced fungicide use—are making AF contamination an increasing concern in the Northern Hemisphere [11]. AFs are produced by fungi of the *Aspergillus* of the section *Flavi*, primarily *Aspergillus flavus* and *Aspergillus parasiticus*. These fungi can contaminate crops both pre-harvest and post-harvest, including during storage and transport [12,13]. AF contamination is reported globally in numerous agricultural crops, particularly cereals such as rice, maize, sorghum, and wheat, as well as in nuts (e.g., peanuts, pistachios, and tree nuts), dried fruits, and cottonseeds [14,15]. Depending on their end use, these crops may enter the food or animal feed chains, each presenting distinct risks to human and animal health. Contaminated crops can be consumed directly by humans or used as livestock feed, allowing AF to enter the food chain indirectly. The main AFs include B1, B2, G1, G2, and M1, with aflatoxin B1 (AFB1) recognized as the only naturally occurring human carcinogen. In addition to posing serious health risks, AF contamination leads to significant economic losses through reduced crop yields and trade disruptions, underscoring the need for continued research on mycotoxin exposure risks [16,17,18].

**Figure 1:**
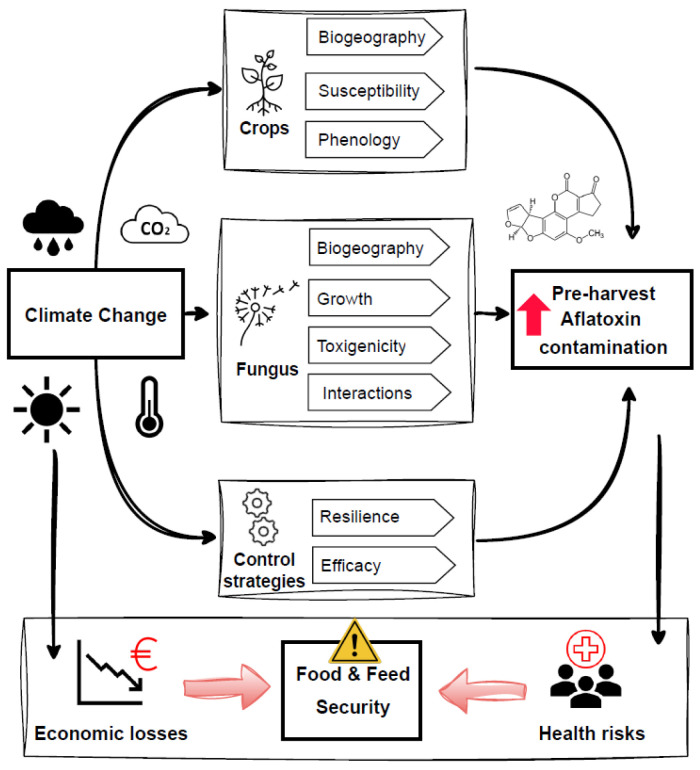
Schematic diagram illustrating the pathways through which climate change influences pre-harvest aflatoxin (AF) contamination in crops. The figure highlights key environmental stressors (e.g., rising temperatures, drought), their interactions with crops, fungus, and control strategies, and the downstream effects on food and feed security.

Climate-related environmental factors significantly influence the colonization of crops by AF-producing fungi, posing serious challenges for FFS and security [19] and affecting AF contamination both pre- and post-harvest [20] (Figure 1). Traditionally, AF-producing fungi have thrived in warm, humid, or drought-prone tropical and subtropical climates [21], which favor the growth of mycotoxin-producing fungi in crops. Extreme weather events, such as droughts and floods, further increase contamination risk by disrupting food storage, processing, and distribution [22,23,24]. However, climate change, including rising temperatures, altered precipitation patterns, increased atmospheric CO_2_, and more frequent extreme weather events, is contributing to heightened AF contamination in parts of southern and eastern Europe [25,26,27,28,29,30,31,32,33]. AF contamination results from a complex interplay of agronomic practices, environmental factors, and climate (Figure 1). Agronomic factors, including sowing and harvest dates, flowering periods, soil conditions, tillage, crop rotation, green manure application, and pest and disease management, play a critical role in pre-harvest AF contamination, particularly in maize [34,35]. Specific conditions, such as the timing of silk emergence, use of resistant maize hybrids, and kernel moisture at harvest, further contribute to the variability in contamination levels across regions and growing conditions [36]. Climate-driven shifts in agronomic practices, such as increased pesticide use resulting from extreme weather and pest pressures, further heighten AF risks [3]. Consequently, new and unpredictable combinations of AF contamination across crops and regions are emerging, making AF control increasingly complex worldwide [37,38]. Predicting the impacts of climate change on AF occurrence globally is therefore critical for effective prevention, supporting both public health and economic stability [23,39]. Efforts to control AF contamination face significant challenges, particularly in developing countries where limited storage infrastructure exacerbates post-harvest contamination. Incidents such as AF contamination in bee pollen in Spain and The Netherlands highlight ongoing gaps in managing pre-harvest contamination [3]. In 2020, the EFSA Panel on Contaminants in the Food Chain (CONTAM) expressed health concerns, concluding that “aflatoxin occurrence should continue to be monitored in the light of potential increases due to climate change using methods with high levels of sensitivity for detection” [5]. In response to growing concerns about climate-related FFS risks, the EU recently updated its regulatory framework through Regulation (EU) 2023/2019, which sets revised maximum levels for aflatoxins in high-risk foodstuffs. This highlights the need for enhanced, climate-informed monitoring systems across Member States to better anticipate and mitigate AF contamination under changing environmental conditions [40].

Given the influence of environmental conditions on fungal growth, predicting AF contamination at the pre-harvest stage is crucial in order to develop timely and effective interventions that reduce health risks and economic losses. Recent advances in predictive modeling have improved our ability to assess AF risk under changing climate scenarios. Mechanistic models like AFLA-maize simulate fungal dynamics by incorporating crop phenology, weather variables, and fungal biology [41], while hybrid frameworks such as PREMA and iCLUE integrate land use, crop distribution, and socio-economic factors for more spatially explicit forecasting [34,42]. Additionally, machine learning approaches, including neural networks and random forests, are being explored for their capacity to model complex environmental interactions and support real-time risk assessments when combined with remote sensing and weather data [43]. These tools are increasingly used in early warning systems across Europe, offering scalable, adaptable, and proactive solutions to manage AF contamination under climate variability.

Previous reviews have examined the influence of weather on AF contamination [44], the interaction between environment, AF production, and biological effects [45], and the application of mathematical models for risk assessment [36]. Recent studies have highlighted climate change-driven mycotoxin risks, including shifts in contamination patterns [46], rising global exposure [38], and increasing AF vulnerabilities linked to expanding maize cultivation in Europe [34]. Bunny et al. (2024) further explore climate change’s impact on AF production, emphasizing temperature and humidity effects, the need for improved models, regulatory challenges, and advances in surveillance technologies [47]. Given the growing concern over climate change-driven AF risks in Europe, such as shifting pre-harvest contamination patterns and increasing vulnerabilities in maize cultivation, we were motivated to review how research on this topic has been conducted across the European region.

Our umbrella review aims to provide a comprehensive overview of peer-reviewed research on pre-harvest AF contamination in relation to climate change-driven environmental factors across Europe. We focus on pre-harvest contamination because it is directly influenced by environmental conditions, including those affected by climate change. Examining contamination at this stage allows us to better anticipate how changing weather patterns, such as temperature fluctuations, humidity levels, and drought, drive colonization of crops by AF-producing fungi. Addressing AF contamination early in the crop cycle is vital, as post-harvest strategies, though effective in regions with advanced storage infrastructure, cannot reverse contamination that has already occurred in the field. Identifying AF risks before storage enables more timely, preventive actions, particularly as climate change introduces new threats across diverse European regions. This review will address the following questions: What insight do European researchers offer on climate-related pre-harvest AF contamination, and what critical data remain missing? How is information presented across studies, and is it sufficiently standardized and comparable? Additionally, we examine which crops are the main focus of AF research and identify the geographical areas in Europe where these studies are concentrated. This synthesis will highlight regional trends and provide a foundation for prioritizing data types and methodologies to ensure consistency and comparability in future research.

## 2. Results

### 2.1. Literature Retrieval

Of the 373 initially identified publications, 333 non-duplicate articles remained. After screening titles and abstracts, 56 were deemed eligible for full-text review. Ultimately, 23 studies met the inclusion criteria for the umbrella review, while 33 were excluded for the following reasons: missing AF occurrence data (*n* = 11), analyzing of storage samples (*n* = 3), ineligible study design (*n* = 10), lack of a link with environmental factors (*n* = 4), or unavailability of the full text (*n* = 5). The PRISMA flow chart and the list of included studies are provided in the Supplementary Material (Figure S1 and Table S1, respectively). A summary of key information from the 23 included articles is presented in Table 1. Supplementary Tables S2 and S3 offer further details on environmental conditions, sampling and analytical methods, and contamination levels of different AF metabolites, also indicating where data were not reported.

### 2.2. Geographical Location and Crops of Included Studies

**Figure 2:**
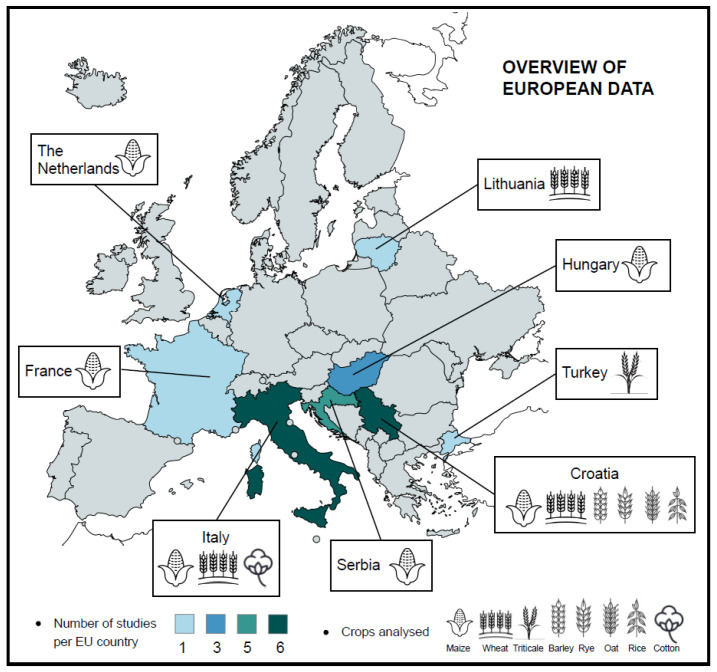
Geographic distribution of studies on pre-harvest aflatoxin (AF) contamination in crops and commodities across European Union countries. The figure shows the number of studies conducted per country and the types of crops analyzed.

Screened peer-reviewed research on pre-harvest AF contamination appears to be concentrated in specific European regions (Figure 2). Most studies were conducted in Italy (*n* = 6), Serbia (*n* = 6), Croatia (*n* = 5), and Hungary (*n* = 3), with one study each from France, The Netherlands, and Lithuania.

Maize dominates AF contamination research in Europe, reflecting its widespread cultivation and critical role in FFS. Studies were conducted in The Netherlands [50], Italy [29,51,61,64], Hungary [52,65,67], Serbia [53,56,57,60,63,68], Croatia [28,55,62,66,68], and France [58].

Wheat, while a globally significant staple, faces lower contamination risks compared to maize but remains a regional focus in Italy [48,54] and Croatia [55,62,66]. Barley and oats are primarily studied in Croatia [55,62,66], reflecting their relatively lower susceptibility to AF contamination and more limited roles in food production. Rice, which is less prone to AF contamination, features in a single study from Turkey [49]. Other regionally specific crops, such as triticale [62], buckwheat [59], cotton [64], and rye [66], have been minimally explored due to their lower levels of cultivation or consumption across Europe.

### 2.3. AF Occurrence and Levels

#### 2.3.1. Contamination Rate

Of the 23 studies included, 90% reported at least one sample exceeding the LOQ, and 16 studies recorded at least one sample surpassing the EU safety threshold. Several studies reported 100% detection rates for specific years, indicating that all tested samples contained AFs [28,48,49,59]. Other studies also revealed high contamination rates, such as Kos et al. (2013) with an average of 30% [53], Leggieri et al. (2015) with 95.6% [29], and Kos et al. (2020) with 94% [60]. In contrast, some studies, including Asselt et al., (2011), Alkadri et al. (2014), and Tóth et al. (2012), reported no detectable contamination, with all samples falling below the LOD [50,52,54]. Moderate contamination rates were observed in studies such as Pietri et al., (2012) at 36% [51], Janić Hajnal et al. (2017) at 57.2% [56], and Kos et al. (2018) at 72.3% [57].

#### 2.3.2. Contamination Level and Uncompliant Samples

Contamination levels varied considerably across the studies. The highest reported AF contamination was observed in maize samples analyzed by Pietri et al. (2012), with a mean of 28.9 µg/kg and a maximum value reaching 1254.1 µg/kg, far exceeding EU safety limits [51]. Similarly high contamination levels were reported by Pleadin et al. (2014), with an average of 81 µg/kg and a maximum of 2072 µg/kg [28], and Kos et al. (2020), with an average of 44 µg/kg and a maximum of 205 µg/kg [60]. These findings highlight the severity of AF presence in certain regions. In most cases, the highest values were recorded for AFB_1_, occasionally for total AF, although the latter was likely driven by AFB_1_ concentrations. In contrast, some studies reported very low contamination levels. For example, Leggieri et al. (2020) observed a mean of 0.275 µg/kg (ranging from 0.1 to 0.4 µg/kg) [61], and Nicolic et al. (2021) reported 3.6 µg/kg (ranging from 0.8 to 8.3 µg/kg) [63].

In Serbia, Kos et al. (2020) found that 94% of maize samples collected in 2012 were contaminated with AFB_1_, with 80% and 61% of samples exceeding the EU regulatory thresholds for human and animal consumption, respectively [60]. A similar pattern emerged in 2015, when AFB_1_ was detected in 90% of maize samples, with 32% [56] and 41% [60] of the samples exceeding the maximum levels permitted for human consumption. In France, AFB_1_ contamination was detected in 6% of field samples and 15% of silo maize samples in 2015 [58].

#### 2.3.3. Mycotoxin Co-Contamination

Co-contamination by multiple *Aspergillus* sections *Flavi* species with varying aflatoxigenic potential, as well as the co-occurrence of multiple AFs, have been reported in Europe [58,60]. In France, *A. flavus* was the most commonly identified *Aspergillus* species in maize samples collected in 2015, representing 69% of the isolates, while *A. parasiticus* accounted for 28%. Among *A. flavus* strains isolated from AF-positive samples, 75% were non-aflatoxigenic, while 24% produced both CPA and AFB. In contrast, 80% of *A. parasiticus* isolates produced both AFB and AFG. Notably, *A. flavus* and *A. parasiticus* were also found in 40% of AF-negative field samples, with half of these isolates identified as toxigenic [58]. In Serbia, Kos et al. (2020) reported frequent co-occurrence of AFs in maize samples collected in 2012, with 71%, 45%, 37%, and 12% of samples co-contaminated with AFB_1_ + AFB_2_, AFB_1_ + AFG_1_, AFB_1_ + AFB_2_ + AFG_1_, and all four regulated AFs, respectively. Additionally, they observed widespread co-contamination with other mycotoxins. Specifically, 94% and 90% of maize samples from 2012 to 2015, respectively, were co-contaminated with AFs and fumonisins (FUMs). In 2015, 59% of samples contained all regulated mycotoxins except OTA [60]. In France, co-contamination with CPA was reported in 28% of field maize samples and 25% of silo samples that also tested positive for Afs [58]. In Italy, mycotoxin co-occurrence in maize was also highlighted and consistently linked to local weather variability and broader climate change influences [61].

### 2.4. Environmental Risk Factors for Contamination

#### 2.4.1. Climate Conditions

##### Weather Parameters

All studies consistently identified rainfall and temperature as the most influential environmental factors affecting AF contamination in crops. The most frequently reported environmental data included measurements of temperature, humidity, and rainfall—particularly the intensity and duration of precipitation—as these variables are key drivers of fungal proliferation and aflatoxin production. Several studies provided specific quantitative weather data, such as mean, maximum, or extreme temperatures, and total rainfall or number of rainy days [28,29,49,51,53,56,57,61,67,68]. In contrast, other studies described weather patterns qualitatively, using general terms such as “dry periods” or “rainy seasons” without providing precise numerical data [48,52,54,55,58,59,60,63,64,65,66].

The temporal scope of weather data varied across studies. Nine studies relied on single-year data to examine the influence of specific climatic events on AF contamination [28,48,49,50,51,56,58,61,62], while fourteen studies used multi-year datasets to capture inter-annual variability and assess long-term climatic trends [29,52,53,54,55,57,59,60,63,64,65,66,67,68].

The granularity of weather data also differed substantially. Some studies provided detailed monthly or even weekly measurements of temperature and rainfall during the growing season, particularly in spring and summer for maize, which allowed for more precise assessments of contamination risks during critical crop development stages such as flowering and harvest [56,57,67,68]. In contrast, other studies presented annual summaries that offered a broader but less detailed view of environmental conditions. In several cases, actual weather data were compared with long-term climatic averages to identify deviations from normal conditions and detect extreme weather events such as droughts or heatwaves [60,68].

**Figure 3:**
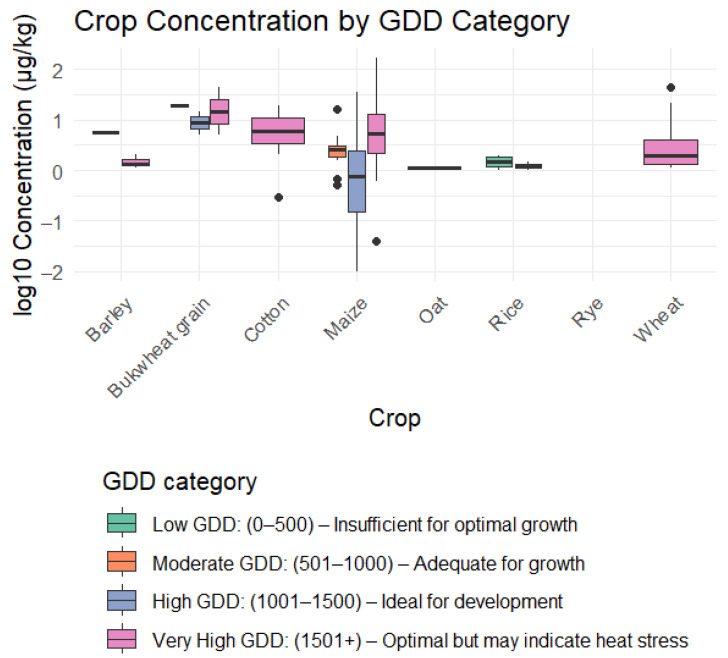
Average aflatoxin (AF) concentrations (µg/kg) reported for different crop types, grouped by Growing Degree Day (GDD) ranges. The figure shows how AF levels change with cumulative heat exposure during the growing season, suggesting a link between temperature accumulation and contamination risk for each crop.

Most studies were conducted during periods characterized by high and very high GDDs, which generally represent optimal conditions for crop development but may signal increased risk of heat stress (Figure 3). Under these thermal conditions, average Afs concentrations, ranging from 10 µg/kg up to 165 µg/kg, were most frequently reported, highlighting this as a critical period for AF development. This pattern was particularly evident in maize, which accounted for the majority of reported outliers.

##### Data Sources and Collection Methods

Most studies relied on secondary data, typically obtained from national or regional meteorological agencies [28,48,49,53,55,56,57,59,60,62,66,68] or directly from nearby meteorological stations [29,50,51,58,61]. Several studies enhanced their analyses by calculating derived indicators such as aridity indices, drought indices, or GDD [50,57,61]. Notably, the study by Molnar et al., (2023) was the only one to use primary data, conducting direct in-situ measurements of air temperature and precipitation [67]. However, 16% of the included studies did not clearly report the sources or collection methods of environmental data, raising concerns about transparency, comparability, and methodological robustness.

#### 2.4.2. Agronomic Factors

Among the 23 studies included in this review, only five provided detailed information on agronomic practices related to pre-harvest AF contamination. Asselt et al. (2011) highlighted the role of phenological conditions in influencing crop development [50]. Keriene et al. (2018) described grain sampling at critical ripening stages (BBCH 77, BBCH 85, and BBCH 89), along with specific milling and storage protocols [59]. Leggieri et al. (2020) examined a range of cropping factors, including phenology, maize hybrid, soil type, tillage practices, and pest management [61]. Nicolic et al. (2021) evaluated mycotoxin contamination across five sweet maize hybrids (PK1, PK3, PK4, PK5, and PK6) [63], while Molnar et al. (2023) detailed maize development from the R2 (Blister) to R6 (Physiological maturity) [67]. The absence of agronomic data in the remaining studies limits the ability to comprehensively assess how pre-harvest agronomic practices influence AF contamination risk.

### 2.5. Methodological Characteristics of Studies

#### 2.5.1. Sampling

##### Origin

The origin of samples across the reviewed studies reflects diverse collection strategies, varying in both geographic scope and the level of aggregation. Several studies aggregated samples collected from provinces, regions, or broader geographic areas to analyze them as combined datasets [29,48,49,53,54,57,64,68]. In contrast, other studies analyzed samples from multiple regions separately, enabling a more detailed examination of spatial variability [28,51,56,61,62,63,65]. A third group of studies focused on specific sampling sites associated with key agricultural stages, such as harvesting locations, mills, or individual farms [50,52,53,55,58,66]. Additionally, experimental field trials provided controlled sampling environments in studies by Keriene et al. (2018), Nicolic et al. (2021), and Molnár et al. (2023) [59,63,67].

##### Timing

The timing of sample collection was reported with varying levels of detail across the reviewed studies. Some studies provided only the year of sampling [28,53,54,66]. Others provided both the year and the specific cropping stage [29,48,50,52,56,58,60,61,62,63,67,68]. A third group offered more detailed information by specifying the year along with either the month or season of sampling [28,49,57,59,64,65]. Notably, Pietri et al. (2012) was the only study to report all three elements: the year, cropping stage, and month or season [51]. Longitudinal studies analyzing data over multiple years offered a broader perspective on temporal trends [28,29,52,57,59,60,63,64,65,66,68].

##### Procedure

The level of detail in sampling descriptions varies considerably across studies, from highly specific methods [66,67] to more generalized approaches [57]. Notably, some studies explicitly followed established guidelines and regulations, such as Commission Regulation (EC) No 401/2006 [40], which sets mandatory EU rules for mycotoxin sampling and analysis in food intended for human consumption [51,56,57,61,68]. Others adhered to international standards like ISO 6497:2002 and ISO 6498:1998, which provide voluntary guidance for feed sampling and preparation, applicable to both quality control and research [28,55]. However, not all studies specified whether their sampling protocols complied with any regulation or standard.

#### 2.5.2. Analysis

##### Number of Samples

The number of samples collected and analyzed varied widely across studies (ranging from 2 to over 10,000), with many not reporting how sample sizes were determined. In most cases, the weight of each laboratory sample also varied considerably between studies and crop types. For instance, Gallo et al. (2018) analyzed 10 grain samples, each weighing approximately 500 g [48], a weight also reported by Kos et al. (2013) and Hajnal et al. (2017) [53,56]. In contrast, Leggieri et al. (2015) prepared 100-g laboratory samples from 10-kg composites [29], while Nicolic et al. (2021) analyzed 2-kg sub-samples [63]. Overall, the level of detail in sampling descriptions differed substantially among studies.

##### Analytical Methods

A range of analytical methods was employed across the screened studies to assess AF levels, reflecting varying degrees of complexity and accuracy. Twelve studies employed high-performance liquid chromatography (HPLC): six used HPLC with fluorescence detection (HPLC-FLD) [29,48,51,56,61,67], and six used HPLC with tandem mass spectrometry (HPLC-MS/MS) [52,54,58,60,62,66]. Seven studies applied the enzyme-linked immunosorbent assay (ELISA) [49,53,55,57,59,63,64]. Additionally, two studies combined ELISA with HPLC MS/MS to enhance reliability [28,68], while one study used the liquid chromatography-mass spectrometry (LC MS/MS) method [50]. One study did not report the analytical method used [65].

##### Detection Limits

In the screened studies, reported LOQs for combined AF components ranged from 0.1 to 50 ug/kg. Many studies also reported LOD; however, the methods used to determine them varied considerably. Several studies, such as Gallo et al. (2008), Asselt et al. (2014), and Keriene et al. (2018), used a signal-to-noise ratio of 3 for conversion between LOQ and LOD [48,50,59]. In contrast, others, such as Pleadin et al. (2015), calculated the LOD by setting the detection threshold at twice the standard deviation of the blank [55].

## 3. Discussion

### 3.1. Scope of the Current Research

#### 3.1.1. Geographical Location and Period Covered

In Europe, AFs contamination in pre-harvest maize was first reported in Italy in 2003, following a long, dry, and hot summer [25]. Geographically, current research remains concentrated in specific European regions, often overlooking areas highly vulnerable to climate change. Southern and Eastern Europe are projected to experience substantial temperature increases and more variable precipitation patterns, yet studies on AF contamination in these regions remain limited. This lack of attention highlights a critical research gap. Expanding research into these at-risk areas could provide valuable insights into emerging AF risks and support regional stakeholders in preparing for potential increases in contamination.

The shortage of peer-reviewed studies on pre-harvest AF contamination across many European countries is concerning and may have serious implications for the food and feed supply chains. This gap hinders effective monitoring and response strategies, making it difficult to assess the true extent of AF-related risks. The growing number of AF incidents, such as the recent warning issued by the Dutch Food and Consumer Product Safety Authority regarding contaminated popcorn sold in an organic supermarket chain, highlights the urgency of addressing this issue. As climate change continues to exacerbate conditions favorable to AF contamination, this research void poses a significant threat to FFS across Europe, with far-reaching consequences for public health and agricultural practices. A deeper understanding of the interaction between climate change and AF risks is essential to inform more effective prevention and mitigation strategies.

#### 3.1.2. Crops

The focus on maize and wheat underscores their agricultural importance; however, other crops also warrant further investigation to better understand their contamination risks under changing climatic conditions. Globally traded crops like groundnuts, peanuts, beans, spices, and coffee, known for their high vulnerability to AF contamination [69,70,71,72], are underrepresented in European studies, likely due to their limited cultivation and consumption in the region. However, climate change may create conditions favorable for growing these crops in Europe, as rising temperatures and longer growing seasons increase regional suitability. While this shift could diversify European agriculture and support local demand, it also raises concerns about AF contamination risks.

Analyzing crop samples intended for both human food and animal feed is essential, as the food and feed chains are closely interconnected. Contamination in one sector can easily affect the other, posing risks to both public and animal health. For instance, AF contamination in feed crops can lead to the presence of AFM1 in milk, directly impacting human health [73]. Since livestock often consume crops also used for human food, contamination can cascade along the food chain, compromising FFS and economic stability. This highlights the importance of a One Health approach, which recognizes the interconnectedness of human, animal, and environmental health. By monitoring both food and feed crops for contaminants such as AFs, we can develop more effective strategies to manage FFS, safeguard public health, and address the emerging risks linked to climate change.

### 3.2. Pre-Harvest AF Contamination in Europe

The results suggested a widespread presence of AF in certain European regions. Several of the reviewed studies reported samples with AF levels above the LOQ, including many exceeding the EU regulatory threshold. These findings appear somewhat inconsistent with occurrence data submitted to EFSA by member states, where only 1–6% of AF data in grains and grain-based products exceeded the LOQ. Nonetheless, EFSA’s independent risk assessment concluded that the calculated margin of exposures for AFB_1_ was below 10,000, an outcome that raises significant health concerns.

### 3.3. Understanding Risk Factors for Pre-Harvest AF Contamination

#### 3.3.1. Climate Conditions

AF contamination was reported under drought conditions or periods of low rainfall, often accompanied by low grain moisture content. In contrast, studies reporting low AF detection rates frequently reported more humid summers or summers with intermittent rainfall. Notably, in France, all the contaminated samples originated from areas most affected by heat and drought, confirming the influence of extreme weather on AF contamination in maize. Severe droughts during the 2012–2015 maize harvests in several European regions, particularly in Serbia and France, were associated with marked increases in AF levels and co-contamination with other mycotoxins. In Serbia, 72% and 37% of maize samples were contaminated with AF in 2012 and 2015, respectively, compared to 0% in 2014 [57]. Similarly, Kos et al. (2020) reported AFB1 contamination in 94% and 90% of maize samples in 2012 and 2015, respectively, versus 0% in 2014 [60]. In France, AFs were found in 6% of samples in 2015, with no prior reports of contamination [58]. This aligns with findings from Italy [23] and Portugal [32], which highlight the heightened AF contamination risks in European maize under climate change scenarios [74].

The influence of environmental factors, particularly rainfall and temperature, on AF contamination in crops is well-documented, with multiple studies consistently highlighting their importance [5,23,74]. However, inconsistencies arise due to variability in the granularity and quality of weather data reported. While some studies provide detailed monthly or weekly measurements, others rely on broader annual summaries, which may mask short-term climatic events critical for fungal growth and toxin production. Additionally, temporal analyses vary widely—some focus on single-year data capturing specific extreme events, whereas others employ longitudinal data to assess inter-annual variability. A significant challenge is the frequent lack of transparency regarding data sources and collection methodologies, which limits reproducibility and hinders robust comparisons. Moreover, although derived environmental indicators such as growing degree-days or aridity indices can offer deeper insights, their inconsistent use and lack of standardization further reduce comparability across studies. These methodological differences contribute to data heterogeneity that complicates meta-analyses and the development of unified risk assessment models, ultimately impacting the formulation of effective, evidence-based policy recommendations.

#### 3.3.2. Agronomic Factors

The limited inclusion of agronomic practices in most studies underscores a critical gap in the current understanding of pre-harvest AF contamination in European crops. Despite evidence that phenology, crop management, and hybrid selection significantly influence AF risk [61,67], these variables are often absent or insufficiently reported. This omission hampers the development of targeted, evidence-based mitigation strategies. The lack of standardized agronomic data also complicates efforts to build predictive models, weakening the reliability of risk assessments across different climatic and cropping contexts.

Furthermore, while some studies acknowledge the importance of pre-harvest control measures—such as biocontrol agents, chemical treatments, and resistant varieties—their effectiveness under future climate conditions is rarely evaluated systematically. This represents a missed opportunity to future-proof mitigation strategies considering increasing abiotic stresses. The dynamic interaction between farming practices and environmental conditions remains poorly explored, despite being critical to understanding real-world contamination scenarios [36,75,76,77]. Additionally, soil health, a potentially influential but under-researched determinant of AF contamination, is another area where the literature falls short [78,79,80,81]. Although emerging evidence suggests that soil pollutants such as dioxins and nanoparticles may exacerbate AF production through oxidative stress and altered fungal metabolism [79,80,81], these mechanisms are rarely integrated into AF risk frameworks. This oversight limits both the ecological validity of existing studies and their practical utility for informing policy.

Taken together, these gaps highlight a broader issue: the fragmented treatment of key contamination drivers across studies. Without more systematic inclusion and harmonized reporting of agronomic and soil variables, cross-study comparisons remain limited, and policy recommendations risk being based on incomplete evidence. Addressing these inconsistencies is essential for advancing both the science and management of AF contamination in European agriculture.

### 3.4. Methodological Shortcomings

#### 3.4.1. Weather Parameters

The findings reveal significant heterogeneity in how weather parameters are reported and analyzed, which poses challenges for the interpretation and comparability of AF contamination studies. While detailed, high-resolution weather data—especially at monthly or weekly intervals—enhance the understanding of contamination dynamics during critical crop growth stages, many studies lack this granularity, potentially obscuring key short-term environmental triggers. Conversely, reliance on aggregated annual data, though useful for identifying long-term trends, often overlooks seasonal anomalies that substantially influence AF risk. Furthermore, the frequent use of qualitative weather descriptors without accompanying quantitative measures reduces the precision and reproducibility of environmental assessments, limiting their application in predictive modeling and risk forecasting. While some studies’ comparisons of observed weather conditions with historical averages provide valuable context for contamination spikes, inconsistent methodologies across studies hinder synthesis and meta-analytic efforts. Collectively, these inconsistencies underline the need for standardized, high-resolution, and quantitatively supported weather data reporting to improve AF risk assessment and enhance cross-study comparability, thereby strengthening the scientific basis for effective policy and management strategies.

#### 3.4.2. Sampling

The sampling process is essential for ensuring reliable and representative results in environmental and agricultural research, directly affecting data quality, interpretability, and comparability. Accurate reporting of sample origin, collection timing, and detailed procedures is essential to fully understand the methodologies employed. The reviewed studies cover diverse geographical regions and agricultural contexts, with sampling periods ranging from single harvest seasons to multi-year longitudinal designs. Longitudinal studies are particularly valuable for detecting trends and maintaining consistency across years. However, methodological transparency varied considerably: while some studies provided robust, well-documented protocols, others lacked critical information on sample origin, timing, or preparation. Notably, several studies included in this umbrella review failed to specify whether grain samples were intended for animal or human consumption. This inconsistency in reporting standards introduces variability in sampling accuracy, increases the risk of bias, and hinders reproducibility. Moreover, without key contextual details, such as the year, cropping stage, and month of sampling, the scientific and societal relevance of results is diminished, limiting their utility beyond regulatory comparisons. These gaps complicate cross-study comparisons and meta-analyses and reduce the potential for aligning findings with FFS policies. To address these limitations, future research should prioritize standardized and transparent documentation of sampling methodologies, thereby enhancing reliability, replicability, and the broader impact of study findings.

#### 3.4.3. Analysis

The diversity of analytical methods used across studies raises important considerations for data quality and comparability in AF assessment. ELISA, used in 7 studies, was often selected due to its simplicity and low cost, but it is not considered a gold standard method, as it has lower specificity and sensitivity compared to chromatographic techniques. In contrast, 12 studies employed chromatographic techniques—6 used HPLC-FLD and another 6 used HPLC-MS/MS. These methods, particularly HPLC-MS/MS, are regarded as gold standard techniques due to their superior analytical accuracy, specificity, and regulatory acceptance. Although a strong correlation between ELISA and HPLC results has been reported [82], the choice of method often depended on sample availability and laboratory resources. Only one study used LC-MS/MS, the most advanced and precise technique currently available. These differences in methodological rigor, including variability in limits of quantification and sample preparation protocols, can contribute to inconsistencies across studies. Therefore, cautious interpretation of results is essential, and future research would benefit from improved data quality and the standardization of analytical methods, as well as the integration of more robust and recent contamination assessments. Recent advances in biosensor technologies have introduced promising alternatives for AF detection, particularly in low-resource or field settings. Among these, electrochemical biosensors based on screen-printed electrodes (SPEs) have gained attention for their affordability, portability, and capacity for rapid, label-free detection [83]. While biosensors are not yet standardized for regulatory use, their integration into early warning systems presents a valuable direction for future AF surveillance strategies.

## 4. Conclusions

The collective findings of this umbrella review underscore the significant impact of climate change on pre-harvest AF contamination in European crops. Rising temperatures, prolonged droughts, and shifting rainfall patterns create conditions that favor aflatoxigenic fungal proliferation and AF production. These changes pose serious risks to food safety, public health, and agricultural stability across Europe. However, the current evidence base is constrained by persistent limitations in data collection, methodological heterogeneity, and reporting practices. Inconsistent study designs, lack of standardized environmental and contamination metrics, and limited geographic and crop coverage reduce comparability across studies and hinder the identification of clear trends or region-specific risk factors.

### 4.1. Practical Implications and Future Research Directions

To improve the reliability, comparability, and practical utility of AF contamination studies under climate change, future research should address the key challenges summarized in Table 2. This table integrates core findings from the literature with corresponding research priorities and actionable recommendations to support harmonized study design, data quality, and policy relevance. Based on this synthesis, several critical needs emerge:

- Geographic expansion of monitoring efforts to include underrepresented and climate-vulnerable regions across Europe.
- Long-term, high-resolution monitoring to capture seasonal variability and long-term trends in climate–AF dynamics.
- Standardized data collection protocols for environmental, crop, and contamination variables, including harmonized sampling methods and clear reporting of AF detection techniques (e.g., LOD/LOQ).
- Broader crop coverage, with inclusion of feed crops and resistant hybrids to support more comprehensive risk assessments.
- Integration of agronomic factors such as sowing and harvest dates, soil characteristics, and field management practices.
- Adoption of a minimal dataset for AF monitoring, including specific variables on climate, crop phenology, sampling, and AF types and levels. See Table S4.
- Development of centralized and accessible databases with standardized metadata and transparent documentation to enable cross-study comparisons and inform evidence-based decision-making.

### 4.2. Final Remarks

Addressing the challenges posed by AF contamination in a changing climate requires coordinated, cross-disciplinary research frameworks that prioritize standardized, transparent, and accessible data collection. By adopting consistent methodologies, integrating comprehensive environmental data, and leveraging advanced analytical tools, future research can generate robust and comparable datasets that provide actionable insights for mitigating AF risks. Establishing centralized, well-structured datasets will not only improve scientific understanding but also support evidence-based policymaking, enabling agricultural systems to adapt effectively to the evolving climate landscape and safeguard food and feed security across Europe.

## 5. Methods

### 5.1. Search Strategy

We conducted an umbrella literature review using the scientific bibliographic databases PubMed and Scopus. We searched for relevant English-language publications up to 14 October 2024. The search strategy was developed based on expert knowledge and a review of key publications. It included both free-text and MeSH terms, covering three main concepts: climate change, AFs, and contamination. These terms were combined using filters to create a comprehensive search string. The full search strategy is available in the Supplementary Material (Table S5).

### 5.2. Selection Criteria

We applied predefined inclusion and exclusion criteria to determine the eligibility of research articles:

- Study type: Only original research articles were included. We excluded reviews, editorials, concept papers, and book chapters.
- Study design: We included case studies and field experiments. Articles based on data modeling, laboratory experiments, or biocontrol trials were excluded.
- Geography: Only studies reporting data from Europe were included.
- Topic: We included studies addressing climate-related changes and pre-harvest AF contamination in crops. Specifically, we included: (a) studies reporting in-field or pre-harvest contamination; (b) studies analysing AFs (i.e., AFB_1_, AFB_2_, AFG_1_, and AFG_2_); and (c) studies investigating climate-related environmental factors (e.g., weather conditions, weather events, and climate patterns). We excluded: (a) studies not addressing either climate change or AFs; (b) studies focused on the health effects of AFs (e.g., toxicology, disease burden); (c) studies analyzing AF occurrence in non-crop samples (e.g., milk, human or animal samples, air, soil); (d) studies reporting mycotoxins other than AFs; (e) studies using artificial inoculation of aflatoxigenic fungi; (f) studies focused solely on post-harvest contamination (e.g., stored, packaged, or marketed products) or environmental factors specific to storage; (g) studies addressing mitigation strategies, analytical methods, or prevention/control measures without considering the role of climate-related environmental factors.
- Results: We included studies that reported quantitative AF occurrence data. Studies reporting only aflatoxigenic fungal colony counts without AF measurements were excluded.
- Language and availability: Only studies published in English with full-text availability were included.

### 5.3. Data Extraction

We applied the predefined eligibility criteria to select full-text articles for inclusion in this umbrella literature review. First, we compiled all articles retrieved from the databases and removed duplicates. Both co-authors, AB and DMF, then independently conducted an initial screening based on titles, keywords, and abstracts, followed by a full-text assessment using the criteria outlined in Section 2.2. The researchers were blinded to each other’s decisions. After full screening, the two authors independently extracted data from the remaining articles on the following items:

- Country: The country where the study was conducted.
- Period: The time frame during which data were collected.
- Environmental conditions: (a) Weather parameters—variables such as temperature, humidity, and rainfall; (b) Data sources—whether the data were primary or secondary; (c) Data collection methods—how environmental data were gathered (e.g., via in-situ measurements, meteorological stations, models, or other databases); (d) Agronomic factors—crop-related conditions such as soil type, irrigation, and farming practices.
- Crop: The specific crop analyzed for AF contamination.
- Sampling: (a) The type(s) of AFs analyzed; (b) The number of samples tested; (c) Analytical methods or techniques used for AF detection (e.g., HPLC, ELISA); (d) Detection limits—the minimum detectable (LOD) and quantifiable (LOQ) concentrations of AFs.
- Contamination: (a) Contamination rate—the percentage of contaminated samples; (b) Contamination level—the measured AF concentrations; (c) Non-compliant samples—the percentage of samples exceeding EU regulatory limits (EU Regulation EC 466/2006) [84].
- Additional information: Any relevant details not covered in the categories above (e.g., policy context, interventions, statistical approaches).

We used Excel to record study selection decisions, data extraction, and management. For data extraction, we applied a self-developed, standardized, and pre-piloted template to facilitate consistency and comparison of information retrieved by both co-authors. The data extracted independently by each co-author were then integrated into a single database and compiled in an Excel spreadsheet.

For visualization purposes, we reclassified the reported weather conditions into Growing Degree Days (GDD), based on the reported temperature, base temperature, average growing days, and crop type. The data used for these calculations are available in the Supplementary Material (GDD calculations, Box S1).

## Figures and Tables

**Table 1:** Summary of key details from studies included in the review, including article reference, sampling strategy, crop type, reported weather parameters, type of aflatoxin (AF) analyzed, contamination rate, and contamination levels.

Article	Crop	Weather Parameters	Contamination		
			Toxin	Rate ^1^	Level ^2^
Gallo et al., 2008 (Italy) [48]	Wheat	2006: May was less rainy and much warmer than usual. June registered a thermal rise and low air humidity levels.	AF	10 (100)	1.1 (0.4–1.9)
Buyukunal et al., 2010 (Turkey) * [49]	Rice	Winter: −7 °C, 70.3% Relative Humidity	AF	25 (100)	1.9 (1.1–3.3)
		Spring: 7.1 °C, 66.7% Relative Humidity	AF	25 (32)	1.7 (<LOD-3.0)
		Summer: 21.9 °C, 47.4% Relative Humidity	AF	25 (28)	1.5 (<LOD-3.0)
		Autumn: 9.8 °C, 57.8% Relative Humidity	AF	25 (100)	1.8 (0.1–3.2)
		All year	AF	100 (65)	1.7 (<LOD-3.2)
Asselt et al., 2011 (The Netherlands) [50]	Maize	Growing degree-days (GDD) as the accumulation of the average daily temperature subtracted from a base temperature of 6 °C, below which maize seeds do not germinate.	AF	42 (0)	<LOD
Pietri et al., 2012 (Italy) [51]	Maize	LR Summer: 22 °C, 41.93 mm	AFB1	66 (36)	28.9 (<LOD-1254.1)
		VR Summer: 22.6 °C, 50.6 mm	AFB1	34 (79)	2.4 (<LOD-29.9)
		P Summer: 20.4 °C, 60.7 mm	AFB1	4 (0)	<LOD
		FV Summer: 21.3 °C, 122.1 mm	AFB1	33 (18)	0.2 (<LOD-3.44)
		E Summer: 23.1 °C, 23.9 mm	AFB1	60 (8)	28.9 (<LOD-1254.1)
Tóth et al., 2012 (Hungary) [52]	Maize	2010 Summer: Rainy	AF	NA (0)	<LOD
		2011 Summer: Dry and hot	AF	NA (0)	<LOD
Kos et al., 2013 (Serbia) [53]	Maize	2012 maize growing season (April–September): extremely hot and dry conditions and drought. N Tmax 30 °C = 68 and 35 °C = 19/Number days precipitation = 39	AF	2009–2011: 60 (0) 2012: 200 (0)	15% of samples had a range of 1–10. 24% from 10 to 50 and 29.5% from 50 to 90
Pleadin et al., 2014 (Croatia) [28]	Maize	2012: was extremely warm (>98%) and dry (<2%), characterized by a very low average rainfall.	AFB1	NC: 460 (40) CC: 97 (28) EC: 633 (38) Total: 633 (38)	NC: 165 (1.2–2072) CC: 34 (1.1–1728) EC: 44 (1.3–945) Total: 81 (1.1–2072)
Alkadri et al., 2014 (Italy) [54]	Wheat	The northern Italian areas have warm, humid summers, with occasional rains compared with the southern part, which is hot and dry.	AF	46 (0)	<LOD
Pleadin et al., 2015 (Croatia) [55]	Maize-M Wheat-W Barley-B, Oat-O	2009, 2011 and 2013: the period of maize planting, growing, and harvesting (April–September) was very to (rarely) extremely warm with normal to scarce precipitation. 2010: was equally warm, but wet to highly wet. 2012: the maize growth and harvesting period (May, August) had warm weather and the lack of precipitation.	AFB1	2009–2013 M: 972 (31) W: 201 (7) B: 147 (6) O: 136 (5)	2009–2013 M: 38.5 ± 75.7 W: 1.6 ± 1.7 B: 1.5 ± 1.2 O: 1.2 ± 0.8
Leggieri et al., 2015 (Italy) * [29]	Maize	2009: Average T in Celsius: 24.1 (21–26)/Humidity, in %: 65 (60–72)/Rain in days: 2.8 (1–6)/Rain in mm: 25.8 (4–59)	AFB1	46 (96)	34.7 ± 115
		2010: Average T in Celsius: 23.5 (22–26)/Humidity in %: 67.6 (59–75)/Rain in days: 4.8 (3–8)/Rain in mm: 76.3 (9–131)	AFB1	48 (77)	15.9 ± 42.9
		2011: Average T in Celsius: 23.2 (21–25)/Humidity in %: 66 (56–73)/Rain in days: 3.7 (0–7)/Rain in mm: 33.8 (0–92)	AFB1	46 (59)	9.8 ± 48.2
Janić Hajnal et al., 2017 (Serbia) * [56]	Maize	NWB 2015 (April–September): N Tmax > 25 °C = 96 and >35 °C = 12/sum Precipitation (mm) = 466	AF	32 (34)	6.7 (1.3–28.1)
		NNB 2015 (April–September): N Tmax > 25 °C = 104 and >35 °C = 23/sum Precipitation (mm) = 292	AF	25 (64)	9.4 (1.4–33.8)
		NSB 2015 (April–September): N Tmax > 25 °C = 105 and >35 °C = 26/sum Precipitation (mm) = 359 (0–92)	AF	90 (64)	11.6 (1.3–91.4)
		CS 2015 (April–September): N Tmax > 25 °C = 110 and >35 °C = 25/sum Precipitation (mm) = 312	AF	33 (91)	18.5 (1.4–86.3)
		All regions 2015: one of the hottest and driest summers in the last ten years in Serbia.	AF	180 (57)	12.7 (1.3–91.4)
Kos et al., 2018 (Serbia) [57]	Maize	2012 (April–September): Extreme drought/N Tmax > 30 °C = 63 and >35 °C = 18/sum P (mm) = 270	AF	600 (72)	37.4 (1.0–111.2)
		2013 (April–September): Dry and hot/N Tmax > 30 °C = 37 and >35 °C = 8/sumP (mm) = 326	AF	600 (25)	13.4 (1.2–65.2)
		2014 (April–September): Rainiest year/N Tmax > 30 °C = 14 and >35 °C = 0/sumP (mm) = 780	AF	600 (0)	<LOD
		2015 (April–September): Dry and hot/N Tmax > 30 °C = 53 and >35 °C = 14/sumP (mm) = 313	AF	600 (37)	9.9 (1.1–76.2)
		2016 (April–September): Moderate weather/N Tmax > 30 °C = 25 and >35 °C = 1/sumP (mm) = 485	AF	600 (5)	3.1 (1.3–6.9)
Bailly et al., 2018 (France) * [58]	Maize	2015: hot and dry climatic conditions during summer (maize flowering period).	AF	118 (6)	20.2 (0.3–70)
Keriene et al., 2018 (Lithuania) [59]	Buckwheat grain	2013: Temperature and amount of rainfall in July–August were close to the long-term average.	AFB1	BBCH85: 12 (0)	BBCH85:<LOD
		2014: The amount of rainfall that fell in August (162.1 mm) was 70% higher than the long-term average.	AFB1	BBCH85: 12 (100) BBCH8: 24 (100)	BBCH85: 14.9 (3.2–25.5) BBCH8: 5.1 (2.2–8.4)
		2015: spring was cold and dry. In June, only 14.1 mm of rainfall (5 times less than the long-term average). In July, rainfall amounted to 75.9 mm. In August, it was dry again.	AFB1	BBCH77: 12 (100) BBCH85: 12 (100) BBCH89: 24 (100)	BBCH77: 43 (1.7–71.6) BBCH85: 19.1 (5.2–33.5) BBCH89: 4.9 (2.9–12.8)
Kos et al., 2020 (Serbia) * [60]	Maize	2012 growing season: was characterized by the highest air temperatures and the lowest amount of precipitation compared to the other years investigated and the long-term average.	AFB1	51 (94)	44 (0.6–205)
		2013 growing season: hot and dry weather conditions were dominant during most of the maize growing season.	AFB1	51 (33)	8 (0.5–48)
		2014 growing season: was characterized by extreme high amount of precipitation.	AFB1	51 (0)	<LOD
		2015 growing season: Hot and dry weather conditions were recorded.	AFB1	51 (90)	8 (0.4–41)
Leggieri et al., 2020 (Italy) [61]	Maize	COL: particularly low AI with the most arid conditions	AFB1	9 (44)	0.275 (0.1–0.4)
		LU: maximum monthly rain. Highest mean AI	AFB1	2 (50)	34 (NA)
		MI: highest mean AI with the widest variability	AFB1	8 (88)	18.1 (0.4–93.8)
		PE: mean AI close to 0	AFB1	5 (80)	3.3 (0.2–12.3)
		ME: mean AI close to 0	AFB1	2 (50)	30.4
		FE: mean AI close to 0	AFB1	8 (0)	<LOD
		COP: mean AI close to 0	AFB1	2 (50)	3.7
		LU: mean AI close to 0	AFB1	5 (40)	2.2 (0.7–3.7)
		MM: particularly low AI with the most arid conditions	AFB1	10 (30)	0.67 (0.5–0.8)
Kifer et al., 2021 (Croatia) * [62]	Maize, Wheat Triticale, Oat, Barley	Gornji Stupnik-GS (control village): The yearly total precipitation was between 853.8 and 888.5 mm	AFB1	20 (0)	<LOD <LOD
		Gunja-G (flooded village): The yearly total precipitation was between 642.7 and 785.5 mm	AFB1	20 (5)	8.2 (NA)
Nikolic et al., 2021 (Serbia) * [63]	Maize hybrid (PK1,3,4,5,6)	2019–2020: The mean monthly temperatures (˃20 °C), total monthly rainfall (>35 mm) and mean monthly relative humidity (RH) (˃50%) at the flowering stage (June) and the milk stage (July) were suitable for fungal maize colonization.	AF	For each year 2019—2020 for ZP & KR PK6:4 (0)	2019—ZP: PK6: 3.0 (NA) 2019—KR: PK6: 4.6 (NA) 2020—ZP: PK6: 1.6 (NA) 2020—KR: PK6: < LOD
Ferrari et al., 2022 (Italy) [64]	Cotton (C), Maize flour (M)	In the present study, it was not possible to combine contamination levels with regional trends and climate patterns. According to Locatelli et al. (2022), 2015 and 2018 are the years in which the highest temperatures of the last 10 years were recorded in Po Valley. The year 2015 especially showed the harshest conditions, with high temperatures (23.46 °C), which were counterbalanced by low rainfall (155.62 mm) with respect to 2018 (209.83 mm).	AFB1	For each year from 2013 to 2020 C: 480 (NA) M: 5278 (NA)	2013: C: 4 (2–6); M: 3 (1.5–5) 2014: C: 5 (3–7.5); M: 3.5 (1.9–5.1) 2015: C: 14 (11.9–16.5); M: 15.6 (14–17) 2016: C:2.1 (0–4.5); M: 12.2 (11–14) 2017: C: 6.9 (4.8–9); M: 6.2 (4.8–8) 2018: C: 18.9 (16.5–21); M: 3.5 (2–5.2) 2019: C: 10 (7.8–12); M: 3.6 (2–5.2) 2020: C: 0.3 (0–2.8); M: 3 (1–4.9)
Mesterházy et al., 2022 (Hungary) [65]	Maize	The weather conditions are warmer and drier in southern counties.	AF	2013: 2009 (NA) 2014: 4743 (NA) 2015: 5713 (NA) 2016: 2010 (NA) 2017: 2107 (NA)	2013: 3.4 (NA) 2014: 1.1 (NA) 2015: 0.3 (NA) 2016: 0.3 (NA) 2017: 1.2 (NA)
Kovač et al., 2022 (Croatia) * [66]	Maize (M), Wheat (W), Barley (B), Rye (R). Oats (O)	2016: May–June, normal to very warm temperature, normal to wet precipitation. August, normal precipitation and temperature. October, low temperatures and high amounts of precipitation	AF	M:61 (0) W:57 (0) B: 2 (0)	M: <LOD W: <LOD B: 5.5 (1.2–9.7)
		2017: May–June, normal to very low temperature, normal to wet precipitation. July—August, extremely high temperatures and drought in the southern regions. October, below-average precipitation.	AFB1 AF AF AF AF	M:23 (9) W:47 (2) B: 7 (0) R: 6 (0) O: 6 (0)	M: 0.5 (NA) W: NA B: <LOD R: <LOD O: <LOD
Molnár et al., 2023 (Hungary) [67]	Maize hybrid (FAO 370–390)	2020: The rainiest growing season with +53.4 mm; the temperature never reached 35 °C.	AFB1	NA	Non irrigated: 0.2 (0.0–1.3) Irrigated: 0.04 (0.0–0.2)
		2021: Extremely dry. Tmax > 35 °C = 3 in R4 during the growing season.			
		2022: The entire growing season was the most severe drought in the area for decades.			
Pleadin et al., 2023 (Croatia) [68]	Maize	2018 April–September: N Tmax > 30 °C = 35 and >35 °C = 0/Number days precipitation = 45/SumP (mm) = 442	AFB1	110 (14)	6.2 (1.6–75.1)
		2019 April–September: N Tmax > 30 °C = 43 and >35 °C = 1/Number days precipitation = 53/SumP (mm) = 615	AFB1	109 (16)	2.5 (1.5–26.9)
		2020 April–September: N Tmax > 30 °C = 31 and >35 °C = 1/Number days precipitation = 47/SumP (mm) = 462	AFB1	103 (19)	1.6 (1.5–3.3)
		2021 April–September: N Tmax > 30 °C = 42 and >35 °C = 8/Number days precipitation = 42/SumP (mm) = 379	AFB1	111 (40)	34.1 (1.5–422.2)
Pleadin et al., 2023 (Serbia) * [68]	Maize	2018 April–September: N Tmax > 30 °C = 42 and >35 °C = 0/Number days precipitation = 54/SumP (mm) = 382	AF	100 (8)	8.1 (NA)
		2019 April–September: N Tmax > 30 °C = 42 and >35 °C = 0/Number days precipitation = 54/SumP (mm) = 382	AF	100 (11)	3 (0.6–10.9)
		2020 April–September: N Tmax > 30 °C = 42 and >35 °C = 0/Number days precipitation = 54/SumP(mm) = 382	AF	100 (5)	2.1 (1.1–3)
		2021 April–September: N Tmax > 30 °C = 42 and >35 °C = 0/Number days precipitation = 54/SumP (mm) = 382	AF	100 (84)	38.8 (0.5–246.3)

* Studies reporting both total aflatoxin (AF) and specific metabolite (AFB1, AFB2, AFG1, AFG2) contamination levels or contamination levels for different crop hybrids. Table 1 presents information prioritized by the authors, primarily on total AF contamination or AFB1 levels (in priority order). Supplementary Table S3 includes additional detailed data on contamination levels. ^1^ Number of total samples (% contaminated samples); ^2^ Mean concentration (Range) or mean concentration ± standard deviation (µg/kg). Abbreviations: LOD, Limit of detection; AF, Aflatoxin; AFB1, Aflatoxin B1; AFB2, Aflatoxin B2; AFG1, Aflatoxin G1; AFG2, Aflatoxin G2; AI, Aridity index; sump, sum of precipitation; N Tmax, Number of days with maximum Temperature; NA, not available; BBCH, Biologische Bundesanstalt, Bundessortenamt und CHemische Industrie.

**Table 2:** Key Challenges, Future Research Directions, and Actionable Recommendations for Standardizing Pre-Harvest Aflatoxin (AF) Contamination Studies under Climate Change.

Key Challenge	Future Research Direction	Actionable Recommendations
Geographic gaps remain	Conduct studies in underrepresented and climate-vulnerable European regions to improve risk mapping and develop localized mitigation strategies.	Expand geographic coverage of AF monitoring studies across diverse agroclimatic zones, especially in currently underrepresented regions.
Short study timelines limit insight	Implement long-term, high-resolution monitoring with frequent sampling intervals to capture seasonal dynamics and long-term climate trends.	Design multi-year studies with regular (e.g., weekly or monthly) sampling to assess temporal patterns and climate anomaly impacts.
Inconsistent and non-standardized data collection	Develop harmonized protocols for collecting and reporting environmental and contamination data to improve cross-study comparability.	Adopt standardized metrics for temperature, humidity, rainfall, and AF levels; ensure clear documentation of methods and data sources (see below).
Narrow crop coverage in studies	Expand research to include a broader range of crops, including feed crops and resistant hybrid varieties, across varying regions.	Include multiple crop types in AF risk assessments; evaluate the performance of resistant hybrids under diverse environmental conditions.
Lack of integration of agronomic and sampling details	Incorporate key agronomic variables and sampling protocols into study designs to strengthen model accuracy and field relevance.	Systematically collect data on sowing/harvest dates, soil conditions, irrigation, and farming practices; align with regulatory sampling standards.
Lack of consensus on essential monitoring variables	Establish a minimal dataset framework encompassing key climate, crop, sampling, and contamination variables for consistent monitoring.	Include in all studies (see Table S4): - Climate: daily temperature (min/max/mean), humidity, rainfall, and drought indices. - Crop: species, phenology, management. - Sampling: dates, locations, methods. - AF: types (B1, B2, G1, G2), concentrations, methods, LOD/LOQ
Inconsistent field study designs hinder comparability	Define best practices for field study designs, including duration, crop selection, and sampling frequency, to improve consistency and data utility.	Follow standardized guidelines for long-term, high-frequency sampling; incorporate both food and feed crops, including resistant varieties.
Fragmented and inaccessible data limit policy application	Promote the development of centralized, open-access databases with standardized metadata and transparent documentation to support integrative analysis.	Ensure data are stored in centralized repositories with clear metadata standards and open access for researchers, policymakers, and stakeholders.

## Data Availability

No new data were created or analyzed in this study. Data sharing is not applicable to this article.

## Supplementary Materials

The following supporting information can be downloaded at: https://www.mdpi.com/article/10.3390/toxins17070344/s1, Figure S1: Search results—PRISMA Flow Diagram; Table S1: List of the studies included the review; Table S2: Summary of additional information on environmental conditions, sampling and analysis across the articles included in the review; Table S3: Summary of detailed data on contamination levels of different AF metabolites across studies included in the review; Table S4: Minimal Dataset and Best Practices for Standardized Monitoring of Pre-Harvest Aflatoxin (AF) Contamination under Climate Change; Table S5: Search string; Box S1: Values for GDseason and Tbase used for Cumulative GDD calculations.

## Glossary

AF	aflatoxin
BEA	Beauvericin
CC	climate change
CPA	Cyclopiazonic Acid
CIT	Citrinin
DON	deoxynivalenol
EA	ergot alkaloids
EFSA	European Food Safety Authority
ELISA	Enzyme-Linked Immunosorbent Assay
ENN	enniatin
FFS	food and feed safety
FUM	fumonisin
GDD	growing degree days
HPLC	High-performance liquid chromatography
IARC	International Agency for Research on Cancer
LC MS/MS	Liquid chromatography mass spectrometry
LOD	limit of detection
LOQ	limit of quantification
OTA	ochratoxin A
ZEA	zearalenone
