# Supplementary material for: Pre-Harvest Aflatoxin Contamination in Crops and Climate Change Factors: A European Overview
Source: Toxins (Basel). 2025 Jul 8;17(7):344. doi: 10.3390/toxins17070344 (PMC12299669; doi:10.3390/toxins17070344)
Supplement: Supplementary file 1 [file toxins-17-00344-s001.zip › toxins-3689174-supplementary.pdf]

Appendix: *Pre-Harvest Aflatoxin Contamination and Climate Change Factors: A European Overview*

Table of contents

Supplementary tables

Table S1. List of the studies included the review.
Table S2. Summary of additional information on environmental conditions, sampling and analysis across the articles included in the review.
Table S3. Summary of detailed data on contamination levels of different AF metabolites across studies included in the review.
Table S4. Minimal Dataset and Best Practices for Standardized Monitoring of Pre-Harvest Aflatoxin (AF) Contamination under Climate Change.
Table S5. Search string.

Supplementary figures

Figure S1. Search results - PRISMA Flow Diagram.

GDD calculations

Box S1. Values for GDseason and Tbase used for Cumulative GDD calculations.

Supplementary tables

Table S1. List of the studies included the review.

Study	Title	Year	Reference (DOI)
Gallo et al., 2008	Mycotoxins in durum wheat grain: Hygienic-health quality of Sicilian production	2008	https://doi.org/10.1111/j.1750-3841.2008.00704.x
Buyukunal et al., 2010	Occurrence of AF, AFB1, OTA in rice commercialized in eastern Turkey.	2010	Not available
Asselt et al., 2011	A Dutch field survey on fungal infection and mycotoxin concentrations in maize.	2011	https://doi.org/10.1080/19440049.2012.689997
Pietri et al., 2012	Mycotoxin levels in maize produced in northern Italy in 2008 as influenced by growing location and FAO class of hybrid.	2012	https://doi.org/10.3920/WMJ2012.1449
Toth et al., 2012	Role of Aspergilli and Penicillia in mycotoxin contamination of maize in Hungary.	2012	https://doi.org/10.1556/AAgr.60.2012.2.5
Kos et al., 2013	Natural occurrence of aflatoxins in maize harvested in Serbia during 2009-2012	2013	https://doi.org/10.1016/j.foodcont.2013.04.004
Pleadin et al., 2014	Aflatoxin B1 occurrence in maize sampled from Croatian farms and feed factories during 2013	2014	https://doi.org/10.1016/j.foodcont.2013.12.022
Alkadri et al., 2014	Natural co-occurrence of mycotoxins in wheat grains from Italy and Syria	2014	https://doi.org/10.1016/j.foodchem.2014.01.052
Pleadin et al., 2015	Annual and regional variations of aflatoxin B1 levels seen in grains and feed coming from Croatian dairy farms over a 5-year period	2015	https://doi.org/10.1016/j.foodcont.2014.07.017
Leggieri et al., 2015	Mycotoxin occurrence in maize produced in Northern Italy over the years 2009–2011: focus on the role of crop related factors	2015	https://doi.org/10.14601/Phytopathol_Mediterr-14632
Janic et al., 2017	Aflatoxins contamination of maize in Serbia: the impact of weather conditions in 2015.	2017	https://doi.org/10.1080/19440049.2017.1331047
Kos et al., 2018	Aflatoxins in maize harvested in the Republic of Serbia over the period 2012-2016.	2018	https://doi.org/10.1080/19393210.2018.1499675
Bailly et al., 2018	Occurrence and identification of aspergillus section Flavi in the context of the emergence of aflatoxins in French maize.	2018	https://doi.org/10.3390/toxins10120525
Keriene et al., 2018	Risk factors for mycotoxin contamination of buckwheat grain and its products	2018	https://doi.org/10.3920/WMJ2018.2299
Kos et al., 2020	Mycotoxins in maize harvested in Republic of Serbia in the period	2020	https://doi.org/10.1080/15569543.2018.1532964

	2012-2015. Part 1: Regulated mycotoxins and its derivatives.		
Leggieri et al., 2020	The impact of seasonal weather variation on mycotoxins: Maize crop in 2014 in northern Italy as a case study.	2020	https://doi.org/10.3920/WMJ2019.2475
Kifer et al., 2021	Fungi and their metabolites in grain from individual households in Croatia.	2021	https://doi.org/10.1080/19393210.2021.1883746
Nicolik et al., 2021	The occurrence of mycotoxins in sweet maize hybrids.	2021	https://doi.org/10.2298/GENSR2103311N
Ferrari et al., 2022	An Eight-Year Survey on Aflatoxin B1 Indicates High Feed Safety in Animal Feed and Forages in Northern Italy.	2022	https://doi.org/10.3390/toxins14110763
Mesterhazy et al., 2022	Mycotoxin contamination of maize (Zea mays L.) samples in Hungary, 2012-2017.	2022	https://doi.org/10.1007/s42976-022-00258-1
Kovac et al., 2022	Regulated Mycotoxin Occurrence and Co-Occurrence in Croatian Cereals.	2022	https://doi.org/10.3390/toxins14020112
Molnar et al., 2023	The Effect of Environmental Factors on Mould Counts and AFB1 Toxin Production by Aspergillus flavus in Maize.	2023	https://doi.org/10.3390/toxins15030227
Pleadin et al., 2023	Aflatoxins in Maize from Serbia and Croatia: Implications of Climate Change	2023	https://doi.org/10.3390/foods12030548

Table S2. Summary of additional information on environmental conditions, sampling and analysis across the articles included in the review.

Article	Environmental conditions			Sampling		Analysis	
	Data source	Data collection method	Agronomic factors	Key factors	Details	Analytical method	LOD and LOQ
Gallo et al., 2008	Secondary data available in the original article	Original data extracted from the article SIAS. 2005 and 2006. Servizio Informativo Agrometeorologico Siciliano. Available from: http:/www.sias.regione.sicilia.it	NA	P: 2006<br>O: Caltanissetta, Enna, Palermo, Trapani<br>T: During harvest	A composite grain sample was obtained from each batch of wheat arriving at the farm. Five elementary samples (500 g) were collected from different areas of the batch. A hand probe was used since it is the only effective method in a static sampling for obtaining a representative sample from grain in a truck bin or other container. These original samples were combined and accurately mixed to form a global sample representative of each specific wheat batch	HPLC -FLD	LOD (ppb)<br>- AF 0.5
Buyukunal et al., 2010	Secondary data available in the original article	Original data extracted from the Turkish State Meteorological Service.	NA	P: 2005-2006<br>O: Kars, Agri, Erzurum, Igdir, and Ardahan provinces<br>T: Between December 2005 and November 2006	They were collected from the same producers and retailers in each season. A total of 100 rice samples were investigated for toxicological (AF, AFB1, and OTA) and microbiological quality. Samples were placed in sterile jars in 100 g portions and brought to the laboratory immediately. Samples used for toxicological determinations were kept at -5ºC in bags while awaiting analysis.	ELISA	LOD (ug/kg)<br>- AF 0.050<br>- AFB1 0.025
Asselt et al., 2011	Secondary data available in the original article	Original data extracted from the nearest weather station for each field. The research team developed a Growing degree-days indicator based on the primary variables.	Phenological conditions	P: 2010<br>O: 42 commercial maize growers<br>T: During harvest	For each of the 42 farms, a total of 40 maize cobs were collected from a selected maize field. As selection criteria of the farms the vicinity of a weather station (within 15 km) and the farm size were used (only famers with at least four fields of cereals (including maize) were selected). After collection, the	LC-MS/MS	LOD (ug/kg)<br>- AFB 0.30<br>- AFG 0.60<br>LOQ (ug/kg)<br>- AFB 1<br>- AFG 2

					samples were packed in small, meshed nylon nets, labelled and arranged in plastic crates to protect the integrity of the samples during transport. The samples were stored at 4C for a period of 3 days at maximum.		
Pietri et al., 2012	Secondary data available in the original article	Original data (mean daily temperature, daily rainfall) was collected from 40 weather stations located near the sampling regions during the period 1 June to 30 September 2008	NA	P: 2008<br>O: Lombardian (LR), Veneto (VR), Piemonte (P), Friuli Venezia (FV), Emilia (E)<br>T: During delivery of lots in summer	Sampling was performed by expert technicians at storage centres during delivery of lots according to Commission Regulation (EC) No 401/2006 (EC, 2006a). 197 samples were collected, shared among the 5 regions as follows: Lombardia 66, Emilia Romagna 60, Veneto 34, Friuli Venezia Giulia 33 and Piemonte 4. The weight of lots coming from the fields was between 10 and 50 tonnes. Weight and number of the incremental samples was about 100 g and between 40 and 100, respectively, and weight of the aggregate samples was between 4 and 10 kg.	HPLC -FLD	LOD (ug/kg)<br>- AFB1 0.04<br>- AFB2 0.02<br>- AFG1 0.04<br>- AFG2 0.02<br>LOQ (ug/kg)<br>- AFB1 0.1<br>- AFB2 0.06<br>- AFG1 0.1<br>- AFG2 0.06
Tóth et al., 2012	Descriptive sentence available in the original article	NA	NA	P: 2010-2011<br>O: 9 and 10 maize growing regions<br>T: samples collected after harvest	NA	HPLC - MS/MS	NA
Kos et al., 2013	Secondary data available in the original article	Original data is extracted from Republic Hydrometeorological Service of Serbia.	NA	P: 2009-2012<br>O: Main maize growing areas in Serbia<br>T: during 2009, 2010, 2011, and 2012	Particular numbers of incremental samples were combined in order to obtain aggregate samples of approximately 2–8 kg. Aggregate samples were homogenized and quartered to obtain a 500 g of laboratory samples which were kept in freezer at 4 °C until the analysis. 500 g of each representative sample	ELISA	LOD (ug/kg)<br>- AF 0.5<br>LOQ (µg/kg)<br>- AF 1

					was ground to a 1 mm particle size using laboratory mill		
Pleadin et al., 2014	Descriptive sentence available in the original article	Original data (descriptive sentence) was collected from the Croatian Meteorological and Hydrological Institute.	NA	P: 2013<br>O: Northern (NC), Central (CC) and Eastern (EC) Croatia<br>T: February to September 2013	Samples were taken from different farms and feed factories. Maize sampling and preparation of the test samples were performed in full line with ISO 6497:2002 and ISO 6498:1998, respectively.	ELISA and HPLC - MS/MS	LOD (ug/kg)<br>- AFB1 1.1
Alkadri et al., 2014	Descriptive sentence available in the original article	NA	NA	P: 2009-2010<br>O: Emilia-Romagna, Toscana, Marche, Umbria, Lazio, Basilicata & Sicilia areas.<br>T: 2009 and 2010 seasons	Forty-six samples (46) of durum wheat were collected. Three incremental samples of at least 1 kg were collected to obtain an aggregate sample of 3 kg total weight. After homogenization, samples were packed in a plastic bag and kept at −20 °C in a dark and dry place until analysis. Just before analysis, a subsample of 200 g was mixed thoroughly to obtain wheat flour.	HPLC-MS/MS	LOD (ug/kg)<br>- AFB1 0.2<br>- AFB2 0.25<br>- AFG1 0.25<br>- AFG2 0.25<br>LOQ (ug/kg)<br>- AFB1 1<br>- AFB2 1<br>- AFG1 1<br>- AFG2 1
Pleadin et al., 2015	Descriptive sentence available in the original article	Original data extracted from the Croatian Meteorological and Hydrological Institute (http://klima.hr/ocjene_arhiva.php).	NA	P: 2009, 2010, 2011, 2012, 2013<br>O: Different farms and feed factories in 4 Croatian regions<br>T: over a 5-year period	The samples were sampled and prepared in full line with the ISO 6497:2002 and ISO 6498:1998, respectively. The prepared test portions were ground to a particle size of 1.0 mm to obtain a fine, non-dried powder, and stored at +4 °C prior to analysis.	ELISA	LOD (mg/kg)<br>- AGB1 1<br>LOQ (mg/kg)<br>- AFB1 1.7
Leggieri et al., 2015	Secondary data available in the original article	Original data obtained from two meteorological stations located in the provinces of Piacenza and Ferrara, Northern Italy	NA	P: 2009-2011<br>O: Northern Italy: provinces of Bologna, Ferrara, Modena, Piacenza, Parma, Ravenna and Reggio Emilia, located in the Emilia Romagna region.<br>T: During the combine harvesting discharge.	One hundred sub-samples (approx. 100 g each) were collected from the kernel flux, and these contributed to the final sample. The final sample (approx. 10 kg) was sent to the laboratory, where a lab-sample of 100 g was randomly taken from each sample for the determination of fungal incidence. The remaining sample was finely ground using a cyclone hammer mill. For each	HPLC -FLD	LOD (ug/kg)<br>- AF 0.05<br>LOQ (ug/kg)<br>- AF 0.15

					sample, 2 kg was stored at -20 °C until analysis for mycotoxins.		
Janić Hajnal et al., 2017	Secondary data available in the original article	Original data provided by the Republic Hydro-Meteorological Service of Serbia.	NA	P: 2015<br>O: Northern Serbia, including Western Backa region (NWB), North Banat region (NNB), and South Banat region (NSB)), and Central Serbia region (CS)<br>T: samples were taken after harvest	Examined maize samples in this study were provided from traders. Sampling was performed by official controllers according to the EU requirements (European Commission 2006b) in order to overcome irregular mycotoxins distribution. Incremental samples were combined in aggregate samples of approximately 5-10 kg. Aggregate samples were homogenized and quartered to obtain a 500 g of laboratory samples which were refrigerated at -20 °C until the analysis.	HPLC – FLD	LOD (ug/kg)<br>- AFB1 0.4<br>- AFB2 0.2<br>- AFG1 0.4<br>- AFG2 0.6<br>LOQ (ug/kg)<br>- AFB1 1.3<br>- AFB2 0.6<br>- AFG1 1.4<br>- AFG2 1.8
Kos et al., 2018	Secondary data available in the original article	Original data provided by the Republic Hydrometeorological Service of Serbia.	NA	P: 2012-2016<br>O: Regions from Northern (Autonomous Province of Vojvodina) and Central Serbia<br>T: every year, from October to April.	Maize sampling was performed in accordance with Commission Regulation 401/2006 (European Commission 2006a) as well as the Serbian Regulation (1988). After collection, maize samples were transported to the laboratory of the Institute of Food Technology, University of Novi Sad. Total amount of aggregate samples (5–10 kg) was homogenised.	ELISA	LOD (ug/kg)<br>- AF 0.5<br>LOQ (ug/kg)<br>- AF 1
Bailly et al., 2018 – Data from farm fields	Primary data available in the original article	Original data calculated from spatialized climatic data from nearly 700 weather stations distributed throughout metropolitan France.	NA	P: 2015<br>O: several farm fields in France<br>T: at harvest	A total of 118 farm fields planted with maize were sampled: at harvest, the farmer was asked to prepare samples respecting the following recommendations. (a) Avoid sampling the margins of the	HPLC - MS/MS	LOD (ug/kg)<br>- AFB1 0.1<br>- AFB2 0.1<br>- AFG1 0.12<br>- AFG2 0.25<br>LOQ (ug/kg)

					field, (b) avoid static sampling of grain, and (c) sample moving grains during three different periods of emptying of the combine harvester. In this way, three different subsamples, each weighing at least 1 kg, were manually collected from the moving grains during harvest. These three subsamples were then combined to obtain a 3 kg final sample from each farm field.		- AFB1 0.25<br>- AFB2 0.25<br>- AFG1 0.25<br>- AFG2 0.5
Bailly et al., 2018 – Data from silos	Primary data available in the original article	Original data calculated from spatialized climatic data from nearly 700 weather stations distributed throughout metropolitan France.	NA	P: 2015<br>O: several farm fields in France<br>T: at harvest	A total of 225 samples were collected in silos belonging to storage companies, cooperatives and private merchants located in 33 French administrative departments. At harvest time, three elementary dried samples were taken before storage at different sampling dates (beginning, middle, and end of harvest period). The resulting 225 samples were therefore representative of the different silos. The elementary samples were then mixed to prepare 81 mixed samples representative of each department, each weighing at least 3 kg. AF contents were measured in these 81 mixed samples.	HPLC – MS/MS	LOD (ug/kg)<br>- AFB1 0.1<br>- AFB2 0.1<br>- AFG1 0.12<br>- AFG2 0.25<br>LOQ (ug/kg)<br>- AFB1 0.25<br>- AFB2 0.25<br>- AFG1 0.25<br>- AFG2 0.5
Keriene et al., 2018	Secondary data (as Figures) available in the original article	Original data provided by the Lithuanian Hydrometeorological Service under the Ministry of Environment, Varėna weather station and by the weather station of the Lithuanian Research Centre for Agriculture and Forestry, Perloja Experimental Station.	Phenological development stages BBCH according to Meier (1997).	P: 2013-2015<br>O: Lithuanian Research Centre for Agriculture and Forestry, Perloja Experimental Station (Varėna district)<br>T: at different phenological development stages BBCH: BBCH 77 – 80% of seed ripe, BBCH 85 – seeds show fully-	The grains of BBCH 77 and BBCH 85 ripening stages were collected manually and at full ripening (BBCH 89) were harvested mechanically. A total of 250 g of each representative sample (12% moisture) was ground in an IKA A11 Basic mill and stored at +4 °C until analysis. Samples of buckwheat flour, bran and hulls were prepared.	ELISA	LOD (ug/kg)<br>2013<br>- AFB1 0.5<br>2014<br>- AFB1 1<br>2015<br>- AFB1 1

				ripe colour, BBCH 89 – harvested product.			
Kos et al., 2020	Secondary data available in the original article	Original data for Northern Serbia for the entire period of maize growing season (April-September) in four different years (2012–2015) provided from the Republic Hydrometeorological Service of Serbia.	NA	P: 2012-2015<br>O: Northern Serbia (Autonomous Province of Vojvodina).<br>T: in the period of four years from 2012-2015 after harvest.	A total of 204 maize samples were collected. Most commercial maize hybrids, currently grown in Northern Serbia, were included in this study. Maize samples were selected to be representative for every investigated year, which means that maize samples were systematically taken from the entire investigated area. Particular numbers of incremental samples were combined in order to obtain aggregate samples.	HPLC - MS/MS	LOD (ug/kg)<br>- AFB1 0.25<br>- AFB2 0.4<br>- AFG1 0.3<br>- AFG2 0.8
Leggieri et al., 2020	Secondary data available in the original article	Original data for Emilia Romagna region from May to September 2014 collected from all data sources available (meteorological stations and radar).	Cropping data: phenology (silk emergence and harvest dates), maize hybrid, preceding crop, soil type, tillage, mineral nutrition, pest and disease control, irrigation, severity of European Corn Borer attack and kernel moisture at harvest.	P: 2014<br>O: Colorno (COL), Luzzara (LU), Mirandola (MI), S.G. Persiceto (PE), Medicina (ME), Guarda Ferrarese (FE), Copparo (COP) Lugo (LU), Maiano Monti (MM)<br>T: at harvest, during combine discharge.	Maize field sampling was performed in 51 fields at harvest, during combine discharge, according to European Commission Regulation (EU) 401/2006 (EC, 2006c). One hundred subsamples (around 100 g each) were collected from the kernel flux and they all contributed to a final sample, around 10 kg for each field, sent to the laboratory within 24 h.	HPLC -FLD	LOD (ug/kg)<br>- AF 0.05<br>LOQ (ug/kg)<br>- AF 0.15
Kifer et al., 2021	Secondary data	Original data was taken for the period 2015–2017 for the Zagreb County and	NA	P: 2016-2015	Samples of stored grain were collected in individual households. A	HPLC - MS/MS	NA

	available in the original article	Vukovar-Srijem County from the Croatian Meteorological and Hydrological Service (https://meteo.hr/index_en.php).		O: Gornji Stupnik (Zagreb County) and Gunja (Vukovar-Srijem County)<br>T: at storage in 2016-2017. The grains, were harvested the year before collection	total of 20 grain samples at each location were collected.		
Nicolic et al., 2021	Secondary data available in the original article (not numbers but Figures)	NA	Mycotoxin contamination of maize kernels was evaluated in five sweet maize hybrids (PK1, PK3, PK4, PK5, PK6).	P: 2019-2020<br>O: Zemun Polje (ZP) and Krnješevci (KR) regions<br>T: at the milk stage of the endosperm development, 23-25 days after silking.	Both locations were experimental fields: the experimental field of Maize Research Institute (Zemun Polje, Belgrade-Zemun) and the experimental field Krnješevci. The grain analysis was performed with 2 kg sub-samples drawn from the primary sample. The primary sample was obtained by mixing smaller samples, taken from several different places of one location, into one sample.	ELISA	LOD (ug/kg) - AF 2
Ferrari et al., 2022	Descriptive sentence available in the original article	Original data (presented as descriptive sentence) was obtained from a previous article by Locatelli et al. (2022)<br><br>https://doi.org/10.3390/toxins14080520	NA	P: 2013-2020<br>O: Northern Italy (Po Valley) collected by the Regional Breeders Association of Lombardy.<br>T: Between September 2013 and July 2021	A total of 10,280 samples were collected from the Breeders of Lombardy and stored in plastic bags before analysis. Samples with moisture content higher than 15% were dried in a stove at 60 ºC for 40 h and subsequently grounded with a knife mill with a 1 mm sieve before AFB1 analysis	ELISA	LOD (ug/kg) - AFB1 1.5<br>LOQ (ug/kg) - AFB1 2
Mesterházy et al., 2022	Descriptive sentence available in the original article	NA	NA	P: 2013-2017<br>O: Bács-Kiskun, Baranya, Békés, Borsod-Abaúj-Zemplén, Fejér, Győr-Moson-Sopron, Hajdú-Bihar, Jász-Nagykun Szolnok, Komárom-Esztergom, Pest, Somogy, Szabolcs-Szatmár-Bereg, Tolna, Vas, Veszprém, Zala regions<br>T: during harvest	The samples originated from farmers from all counties of Hungary who sent their grain samples for mycotoxin determination. Most of the samples were mixed. A significant part of the toxin concentration determinations of the grains harvested in September, October or later was performed next year when it was sold or used. There is no information regarding the length of the storage period, and because of this, the preharvest or	NA	NA

					postharvest character of the toxin contamination is not specified.		
Kovac et al., 2022	Descriptive sentence and Heat Maps available in the original article	Original data (presented as Heat Maps and a descriptive sentence) obtained from the Croatian Meteorological and Hydrological Service.<br>years 2016 and 2017.	NA	P: 2016-2017<br>O: Warehouses and silos in Croatian fields<br>T: during the years 2016-2017	A total of 209 samples of unprocessed cereals (maize 84, wheat 104, barley 9, rye 6 and oats 6) were sampled. The samples were collected from all Croatian counties, at least one sample from each county and the rest according to the cereal type of representation.	HPLC - MS/MS	LOD (ug/kg)<br>- AFB1: 0.3<br>- AFB2: 0.15<br>- AFG1: 0.3<br>- AFG2: 0.15<br>LOQ (ug/kg)<br>- AFB1: 1.0<br>- AFB2: 0.5<br>- AFG1: 1.0<br>- AFG2: 0.5
Molnár et al., 2023	Primary data available in the original article	Primary data was collected by the research team with a standard weather station deployed outside the plots at 50 m from the experiment.	Maize growth stages: R2 Blister, R3 Milk, R4 Dough, R5 Dent, R6: Physiological maturity.	P: 2020-2022<br>O: Hungary at the Experimental Station of the Centre of Agricultural Sciences, University of Debrecen.<br>T: The experiment was carried out during the 2020/2022 growing season.	A complex small-plot field experiment was conducted on chernozem soil. Dried kernels were grounded and collected in sterile Stomacher homogenizer bags, homogenized and total mould counts were determined on CYG Agar medium. Inoculated solid agar media were incubated at 30 ºC for five days for mould count determination. All inoculations were done in triplicate.	HPLC -FLD	NA
Pleadin et al., 2023 – Croatian data	Secondary data available in the original article	Primary data provided by the Croatian Meteorological and Hydrological Service.	NA	P: 2018-2021<br>O: Maize producers from four Croatian regions (Central Croatia, Eastern Croatia, Northern Croatia and Western Croatia).<br>T: Immediately after the process of maize drying in dryers and before their storage in the capacities of the manufacturers or further distribution to domestic industries.	The samples were taken from the same localities (production areas) each year dependent of the production capacities. They were obtained directly from farmers or medium size family enterprises. Sampling and sample preparation were performed fully in line with the provisions of the Commission Regulation No. 401/2006.	ELISA and HPLC – MS/MS (in samples > 5 ug/kg AFB1)	LOD (ug/kg)<br>- AFB1 0.2<br>- AFB2 0.25<br>- AFG1 0.25<br>- AFG2 0.25<br>LOQ (ug/kg)<br>- AFB1 1<br>- AFB2 1<br>- AFG1 1<br>- AFG2 1

Pleadin et al., 2023 – Serbian data	Secondary data available in the original article	Primary data provided by the Republic Hydrometeorological Service of Serbia.	NA	P: 2018-2021<br>O: Northern Serbia (Backa, Banat, Srem)<br>T: Depending on the moisture content, maize samples were taken immediately after harvesting from farmers (moisture content 12–14%) or after drying in dryers (moisture content after harvest > 14%).	Maize sampling was conducted by official controllers in accordance with the rules stipulated by the Serbian and European Union Regulations. Depending on the moisture content, maize samples were taken from farmers or dryers, before storage in the producer's facilities or further distribution.	HPLC - MS/MS	LOD (ug/kg)<br>- AFB1 0.2<br>- AFB2 0.25<br>- AFG1 0.25<br>- AFG2 0.25<br>LOQ (ug/kg)<br>- AFB1 1<br>- AFB2 1<br>- AFG1 1<br>- AFG2 1

Abbreviations: P, Period under study; O, Origin of the samples; T, Timing of the sampling; LOD, Limit of detection; LOQ, Limit of quantification; AF, Aflatoxin; AFB1, Aflatoxin B1; AFB2, Aflatoxin B2; AFG1, Aflatoxin G1; AFG2, Aflatoxin G2; ELISA, Enzyme-Linked Immunosorbent Assay; HPLC – MS/MS, High-performance liquid chromatography with two mass spectrometry detectors; HPLC - FLC, High-performance liquid chromatography with fluorescence detector; MSPD, matrix solid phase dispersion; NA, not available; BBCH, Biologische Bundesanstalt, Bundessortenamt und CHemische Industrie;

Table S3. Summary of detailed data on contamination levels of different AF metabolites across studies included in the review.

Article	Sampling	Crop	Weather parameters	Contamination		
				**Toxin**	**Rate [1]**	**Level [2]**
Buyukunal et al., 2010 (Turkey)	P: 2005-2006<br>O: Kars,Agri, Erzurum, Igdir, and Ardahan provinces<br>T: Between December 2005 and November 2006	Rice	Winter: -7ºC, %70.3 Relative Humidity	AFB1	25 (48)	1.32 (<LOD-1.85)
			Spring: 7.1ºC, %66.7 Relative Humidity	AFB1	25 (28)	1.02 (<LOD-1.86)
			Summer: 21.9ºC, %47.4 Relative Humidity	AFB1	25 (20)	1 (<LOD-1.70)
			Autumn: 9.8ºC, %57.8 Relative Humidity	AFB1	25 (44)	1.14 (<LOD-1.76)
			All year	AFB1	100 (35)	1.12 (<LOD-1.86)
Leggieri et al., 2015 (Italy)	P: 2009-2011<br>O: Northern Italy: provinces of Bologna, Ferrara, Modena, Piacenza, Parma, Ravenna and Reggio Emilia, located in the Emilia Romagna region.<br>T: During the combine harvesting discharge.	Maize	2009: Average T in Celsius: 24.1 [21-26] / Humidity (%): 65 [60-72] / Rain (days): 2.8 [1-6] / Rain (mm): 25.8 [4-59]	AFB2<br>AFG1<br>AFG2	46 (72)<br>46 (30)<br>46 (13)	2.2 ± 7.1<br>1.4 ± 4.6<br>0.1 ± 0.2
			2010: Average T in Celsius: 23.5 [22-26] / Humidity (%): 67.6 [59-75] / Rain (days): 4.8 [3-8] / Rain (mm): 76.3 [9-131]	AFB2<br>AFG1<br>AFG2	48 (54)<br>48 (41)<br>48 (15)	1.5 ± 4.1<br>1.8 ± 4.6<br>0.1± 0.2
			2011: Average T in Celsius: 23.2 [21-25] / Humidity (%): 66 [56-73] / Rain (days): 3.7 [0-7] / Rain (mm): 33.8 [0-92]	AFB2<br>AFG1<br>AFG2	46 (28)<br>46 (15)<br>46 (4)	0.7 ± 3.5<br>0.7 ± 3.5<br>< 0.1
Janić Hajnal et al., 2017 (Serbia)	P: 2015<br>O: Northern Serbia, including Western Backa region (NWB), North Banat region (NNB), and South Banat region (NSB)), and Central Serbia region (CS)<br>T: samples were taken after harvest	Maize	NWB Growing season 2015 (April-September):<br>N Tmax>25ºC = 96<br>N Tmax >35ºC = 12<br>sum Precipitation (mm) = 466	AFB1<br>AFB2<br>AFG1<br>AFG2	32 (34)<br>32 (3)<br>32 (3)<br>32 (0)	5.3 (1.3-13.1)<br>1.1(1.1-1.1)<br>13.9 (13.9-13.9)<br><LOD
			NNB Growing season 2015 (April-September):<br>N Tmax >25ºC = 104<br>N Tmax >35ºC = 23<br>sum Precipitation (mm) = 292	AFB1<br>AFB2<br>AFG1<br>AFG2	25 (64)<br>25 (20)<br>25 (4)<br>25 (0)	8.8 (1.4-32.3)<br>1.00 (0.61-1.5)<br>4.4 (4.4-4.4)<br><LOD
			NSB Growing season 2015 (April-September):<br>N Tmax >25ºC = 105<br>N Tmax >35ºC = 26<br>sum Precipitation (mm) = 359	AFB1<br>AFB2<br>AFG1<br>AFG2	90 (51)<br>90 (7)<br>90 (7)<br>90 (4)	10.3 (1.3-88.8)<br>1.5 (0.66-2.8)<br>5.9 (1.8-14.4)<br>3.8 (2.2-7.5)
			CS Growing season 2015 (April-September):<br>N Tmax >25ºC = 110<br>N Tmax >35ºC = 25<br>sum Precipitation (mm) = 312	AFB1<br>AFB2<br>AFG1<br>AFG2	33 (91)<br>33 (39)<br>33 (6)<br>33 (3)	16.7 (1.4-63.5)<br>1.3 (0.60-2.4)<br>16.1 (3.8-28.5)<br>4.1 (4.1-4.1)
			All regions Growing season 2015: The summer in 2015 was one of the hottest summers in the last ten years in Serbia with very little precipitation.	AFB1<br>AFB2<br>AFG1<br>AFG2	180 (57)<br>180 (14)<br>180 (6)<br>180 (3)	11.4 (1.3–88.8)<br>1.3 (0.60 – 2.8)<br>8.6 (1.8 -28.5)<br>3.8 (2.1 – 7.5)
Bailly et al., 2018 (France)	P: 2015<br>O: several farm fields in France	Maize	2015: hot and dry climatic conditions during summer (maize flowering period).	AFB1<br>AFG1	118 (6)<br>118 (6)	12.23 (0.1–66)<br>5.5 (0.2–24.8)

	T: at harvest					
Kos et al., 2020 (Serbia)	P: 2012-2015<br>O: Northern Serbia (Autonomous Province of Vojvodina).<br>T: in the period of four years from 2012-2015 after harvest.	Maize	<u>2012 growing season</u>: was characterized by the highest air temperatures and the lowest amount of precipitation compared to the other years investigated and the long-term average.	AFB2<br>AFG1<br>AFG2	51 (71)<br>51 (45)<br>51 (12)	5 (0.7-22)<br>10 (0.4-141)<br>16 (2-73)
			<u>2013 growing season</u>: hot and dry weather conditions were dominant during most of the maize growing season.	AFB2<br>AFG1<br>AFG2	51 (10)<br>51 (2)<br>51 (0)	1 (0.7-2)<br>3<br><LOD
			<u>2014 growing season</u>: was characterized by extreme high amount of precipitation.	AFB2<br>AFG1<br>AFG2	51 (0)<br>51 (0)<br>51 (0)	<LOD<br><LOD<br><LOD
			<u>2015 growing season</u>: Hot and dry weather conditions were recorded.	AFB2<br>AFG1<br>AFG2	51 (16)<br>51 (14)<br>51 (0)	2 (0.8-2)<br>0.8 (0.3-1)<br><LOD
Kifer et al., 2021 (Croatia)	P: 2016-2015<br>O: Gornji Stupnik-GS (control village) and Gunja-G (flooded village)<br>T: at storage in 2016-2017. The grains, were harvested the year before collection	Maize, Wheat Triticale, Oat, Barley	<u>Gornji Stupnik-GS (control village):</u> The yearly total precipitation was between 853.8 - 888.5 mm	AFB2	20 (0)	<LOD
			<u>Gunja-G (flooded village):</u> The yearly total precipitation was between 642.7-785.5 mm"	AFB2	20 (5)	<LOD
Nicolic et al., 2021 (Serbia)	P: 2019-2020<br>O: Zemun Polje (ZP) and Krnješevci (KR) regions<br>T: Maize kernel samples were collected at the milk stage of the endosperm development, 23-25 days after silking	Maize hybrids (PK1, PK3, PK4, PK5, PK6)	<u>2019-2020</u>: The mean monthly temperatures (>20°C), total monthly rainfall (>35 mm) and mean monthly relative humidity (RH) (>50%) at the flowering stage (June) and the milk stage (July) were suitable for fungal maize colonisation. Climate factors in both growing seasons were similar and favourable for the development of fungal species.	AF	<u>2019 – ZP</u><br>PK1: 4 (0)<br>PK3: 4 (0)<br>PK4: 4 (0)<br>PK5: 4 (0)<br><u>2019 – KR</u><br>PK1: 4 (0)<br>PK3: 4 (0)<br>PK4: 4 (0)<br>PK5: 4 (0)<br><u>2020 – ZP</u><br>PK1: 4 (0)<br>PK3: 4 (0)<br>PK4: 4 (0)<br>PK5: 4 (0)<br><u>2020 – KR</u><br>PK1: 4 (0)<br>PK3: 4 (0)<br>PK4: 4 (0)<br>PK5: 4 (0)	2019 – ZP<br>PK1: 1.538 (NA)<br>PK3: 2.014 (NA)<br>PK4: 2.188 (NA)<br>PK5: 3.463 (NA)<br><u>2019 – KR</u><br>PK1: 2.551 (NA)<br>PK3: 3.051 (NA)<br>PK4: < LOD<br>PK5: 3.27 (NA)<br><u>2020 – ZP</u><br>PK1: 1.84 (NA)<br>PK3: 2.48 (NA)<br>PK4: 2.88 (NA)<br>PK5: 2.74 (NA)<br><u>2020 – KR</u><br>PK1:2.86 (NA)<br>PK3: 2.14 (NA)<br>PK4: 0.67 (NA)<br>PK5: 4.5 (NA)

Kovac et al., 2022 (Croatia)	P: 2016-2017<br>O: Warehouses and silos in Croatian fields<br>T: during the years 2016-2017	Maize (M), Wheat (W), Barley (B), Rye (R). Oats (O)	<u>2016</u>: In May-June temperature conditions were normal to very warm, precipitation conditions were normal to wet. August had normal precipitation and normal to warm temperatures. October the average monthly air temperatures were mostly below the multi-year average and there were high amounts of precipitation above the multi-year average.	AF		
			<u>2017</u>: In May and June temperature conditions were normal to very warm, while precipitation conditions were normal to wet. High temperatures during the silking period, i.e., a very warm July and extremely warm August throughout the country and drought in the southern regions. Below-average precipitation in October.	AFB2<br>AFG1<br>AFG2	M: 23 (4)<br>M: 23 (4)<br>M: 23 (0)	M:1.7 (NA)<br>M: NA (NA)<br>M: 16.22 (NA)
Pleadin et al., 2023 (Serbia)	P: 2018-2021<br>O: Northern Serbia (Backa, Banat, and Srem)<br>T: Depending on the moisture content, maize samples were taken immediately after harvesting (moisture content 12–14%) or after drying in dryers (if moisture content after harvest > 14%). Dependent on that, maize samples were taken from farmers or dryers, before storage in the producer's facilities or further distribution.	Maize	<u>2018</u> Between April-September: N Tmax > 30ºC = 42 / N Tmax > 35ºC = 0 / Number days precipitation = 54 / Sum of precipitation (mm) = 382/ Ratio between sum of precipitation and long-term average (1981-2010) = 106	AFB1<br>AFB2<br>AFG1<br>AFG2	100 (8)<br>100 (0)<br>100 (1)<br>100 (1)	3.6 (0.8-8.3)<br><LOD<br>1.7 (NA)<br>8.1 (NA)
			<u>2019</u> Between April-September: N Tmax > 30ºC = 42 / Number days > 35ºC = 0 / Number days precipitation = 54 / Sum of precipitation (mm) = 382 / Ratio between sum of precipitation and long-term average (1981-2010) = 106	AFB1<br>AFB2<br>AFG1<br>AFG2	100 (11)<br>100 (0)<br>100 (0)<br>100 (0)	3 (0.6-10.9)<br><LOD<br><LOD<br><LOD
			<u>2020</u> Between April-September:<br>N Tmax > 30ºC = 42 / N Tmax > 35ºC = 0 / Number days precipitation = 54 / Sum of precipitation (mm) = 382 / Ratio between sum of precipitation and long-term average (1981-2010) = 106	AFB1<br>AFB2<br>AFG1<br>AFG2	100 (5)<br>100 (0)<br>100 (0)<br>100 (0)	2.1 (1.1-3)<br>< LOD<br>< LOD<br>< LOD
			<u>2021</u> Between April-September: N Tmax > 30ºC = 42 / N Tmax > 35ºC = 0 / Number days precipitation = 54 / Sum of precipitation (mm) = 382 / Ratio between sum of precipitation and long-term average (1981-2010) = 106	AFB1<br>AFB2<br>AFG1<br>AFG2	100 (84)<br>100 (26)<br>100 (20)<br>100 (10)	30.5 (0.5-246.3)<br>3.9 (1.8-13.9)<br>26 (1.2-173.9)<br>7.7 (2.7-30.7)

[1] Number of total samples (% contaminated samples); [2] Mean concentration (Range) or mean concentration ± standard deviation (μg/kg).
Abbreviations: P, Period under study; O, Origin of the samples; T, Timing of the sampling; LOD, Limit of detection; AF, Aflatoxin; AFB1, Aflatoxin B1; AFB2, Aflatoxin B2; AFG1, Aflatoxin G1; AFG2, Aflatoxin G2; AI, Aridity index; sump, sum of precipitation; N Tmax, Number of days with maximum Temperature; NA, not available; BBCH, Biologische Bundesanstalt, Bundessortenamt und CHemische Industrie.

Table S4. Minimal Dataset and Best Practices for Standardized Monitoring of Pre-Harvest Aflatoxin (AF) Contamination under Climate Change

Category	Recommended Variables / Practices	Rationale / Notes
Climatic Data	- Daily max, min, and mean temperature<br>- Relative humidity<br>- Rainfall amount and frequency<br>- Drought indices	Capture key climate drivers influencing fungal growth and AF production
Crop Data	- Crop species and variety<br>- Phenological stage at sampling<br>- Sowing and harvest dates<br>- Irrigation and fertilization practices	Contextualize contamination risk based on crop development and management
Sampling Protocols	- Sampling date and GPS coordinates<br>- Sample size and type (grain, feed, soil)<br>- Sample handling and preparation methods standardized	Ensure reproducibility and comparability of contamination measurements
AF Contamination Data	- AF types measured (B1, B2, G1, G2)<br>- Quantitative concentration levels (μg/kg)<br>- Analytical methods used<br>- Limits of detection and quantification reported	Accurate, consistent AF quantification aligned with regulatory standards
Field Study Design	- Long-term monitoring (multi-year)<br>- High-frequency sampling (weekly/monthly)<br>- Inclusion of diverse crops and resistant hybrids	Capture temporal and spatial variability; broaden risk assessment scope
Agronomic Data	- Soil characteristics (texture, organic matter, pH)<br>- Farming practices (crop rotation, tillage)<br>- Pest and disease management	Integrate factors affecting fungal ecology and AF contamination
Data Management	- Centralized, accessible databases with standardized metadata<br>- Transparent documentation of methods and protocols	Facilitate data sharing, cross-study analyses, and policy application

Table S5. Search string.

Database	Terms	Search strategy
PubMed & Scopus	#1 – Climate Change	("climate change"[MeSH Terms], "climate change" OR "climate warming" OR "climate variation" OR "global warming" OR “climate factors” OR “climatic factors” OR "climatic condition" OR "climatic change" OR "climatic warming" OR "climatic variation" OR "weather" OR "climate scenario" OR "climate impact" OR "agroclimat" OR "drought" OR "flood" OR "abiotic stress" OR "abiotic factor" OR "abiotic condition" OR "temperature stress" OR "temperature impact" OR "temperature effect" OR "effect of temperature" OR "effects of temperature" OR (“increase temperature”[Title/Abstract: ~5]) OR (“warm temperature” [Title/Abstract: ~2]) OR (“warm summer”[Title/Abstract: ~2]) OR (“dry temperature” [Title/Abstract: ~2]) OR (“dry summer” [Title/Abstract: ~2]) OR “solar radiation” OR “co2" OR “moisture” OR "water stress" OR “rainfall” OR “environmental conditions” OR “environmental stress” OR “hot and dry conditions” OR “extreme precipitation”[tiab])
	#2 - Aflatoxin	("aflatoxins"[MeSH Terms], "aflatoxin" OR "flavus" OR "parasiticus" OR "Aflatoxin B1" OR "AFB1" OR "Aflatoxin B2" OR "AFB2" OR "Aflatoxin G1" OR "AFG1" OR "Aflatoxin G2" OR "AFG2" OR "Aflatoxin M1" OR "AFM1" OR "Aflatoxin M2" OR "AFM2" OR "Aflatoxicol" OR "Aflatoxin Q1" OR "AFQ1"[tiab])
	#3 - Contamination	(“contamination" OR "contaminated" OR "contamina" OR “occurrence” OR “concentration” OR “growth” OR “level” OR “presence” OR “prevalence” OR “production” [tiab])
	#4 - Europe	(“United Kingdom" OR "Germany" OR "Spain" OR "Italy" OR "France" OR "Netherlands" OR "Turkey" OR "Poland" OR "Russian Federation" OR "Portugal" OR "Sweden" OR "Finland" OR "Austria" OR "Hungary" OR "Belgium" OR "Denmark" OR "Greece" OR "Romania" OR “Czech Republic" OR "Serbia" OR "Switzerland" OR "Ireland" OR "Croatia"

		OR "Norway" OR "Slovakia" OR "Slovenia" OR "Ukraine" OR "Lithuania" OR "Bulgaria" OR "Estonia" OR "Bosnia and Herzegovina" OR "Latvia" OR "Belarus" OR "Iceland" OR "Cyprus" OR "North Macedonia" OR "Europe" OR "EU" OR "British" OR "German" OR "Spanish" OR "Italian" OR "French" OR "Dutch" OR "Turkish" OR "Polish" OR "Russian" OR "Portuguese" OR "Swede" OR "Finish" OR "Austrian" OR "Hungarian" OR "Belgian" OR "Danish" OR "Greek" OR "Romanian" OR "Czech" OR "Serbian" OR "Swiss" OR "Irish" OR "Croat" OR "Norwegian" OR "Slovak" OR "Slovene" OR "Ukrainian" OR "Lithuanian" OR "Bulgarian" OR "Estonian" OR "Bosnian" OR "Latvian" OR "Belarusian" OR "Icelander" OR "Cypriot" OR "Macedonian" [tiab])
	#5 – Storage	("Storage" OR "storehouse" OR "container" OR "storeroom" [tiab])
	#6 – Post-harvest	("post harvest" OR "post-harvest" [tiab])
	#7 – Climate Change & Aflatoxin contamination	#1 AND #2 AND #3
	#8 – Climate Change & Aflatoxin contamination in Europe	#1 AND #2 AND #3 AND #4
	#9– Complete search	#1 AND #2 AND #3 AND #4 NOT #5 NOT #6 AND (english[Filter])

Supplementary figures

Figure S1. Search results - PRISMA Flow Diagram.

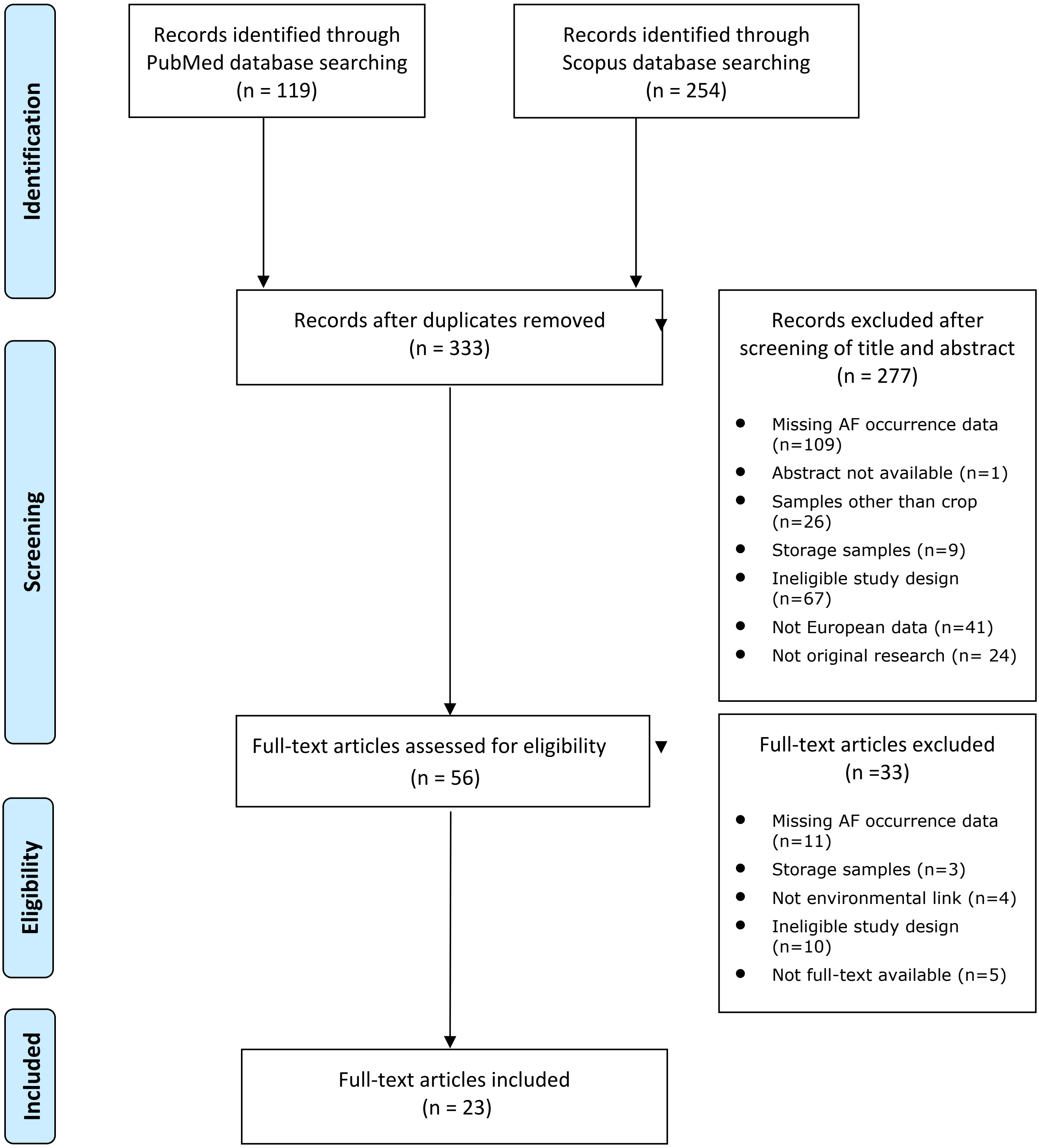

From: Moher D, Liberati A, Tetzlaff J, Altman DG, The PRISMA Group (2009). *P*referred *R*eporting *I*tems for *S*ystematic Reviews and *M*eta-*A*nalyses: The PRISMA Statement. PLoS Med 6(7): e1000097. doi:10.1371/journal. pmed1000097. For more information, visit www.prisma-statement.org.

GDD calculations

In order to represent the reported temperatures and crops in a unified unit, we calculated the GDD (Growing Degree Days). The GDD is a measure used in e.g. agriculture and horticulture to estimate the growth and development of plants during the growing season. It's based on the idea that development only occurs when temperatures are above a certain base temperature for each respective crop.

The GDD formulate used is as follows:

$$Cumulative\ GDD = \left(T_{avg} - T_{base}\right) \times GD_{season}$$

Where: T_{avg} = Average temperature reported by a study; T_{base} = Base temperature for the crop of interest GD_{season} = Average number of days that the growing season takes for each crop type.

The T_{avg} reported by studies was collected from Table 1, while for the studies that did not report any temperature but reported instead "warm" or "very warm" conditions, values of 25 and 30 degrees Celsius were attributed, respectively. For these studies the margin of error when classifying Cumulative GDD is larger, however they would either fall within high or very high GDD classification. The values for Tbase and GDseason were retrieved from available peer-reviewed and grey literature (as available) (See Table S2)

Box S1. Values for GDseason and Tbase used for Cumulative GDD calculations.

Crop	GDseason (Days)	Source
Maize	100	https://doi.org/10.3390/agronomy11030539
Oats	95	https://eos.com/blog/how-to-grow-oats/
Rye	135	https://eos.com/crop-management-guide/rye-growth-stages/
Barley	65	https://eos.com/blog/growing-barley/
Wheat	115	https://eos.com/blog/growing-wheat/
Tritical	105	https://www.saskatchewan.ca/business/agriculture-natural-resources-and-industry/agribusiness-farmers-and-ranchers/crops-and-irrigation/field-crops/cereals-barley-wheat-oats-triticale/triticale-production-and-use
Buckwheat	77	http://www.hort.cornell.edu/bjorkman/lab/buck/guide/growthdevelopment.php
Rice	125	https://eos.com/blog/how-to-grow-rice/
	**Tbase (°C)**	
Maize	10	https://doi.org/10.3390/agronomy11030539
Oats	4.4	https://climate.colostate.edu/gdd.html
Rye	4.4	https://climate.colostate.edu/gdd.html
Barley	4.4	https://climate.colostate.edu/gdd.html
Wheat	4.4	https://climate.colostate.edu/gdd.html
Tritical	5	https://doi.org/10.3168/jds.2018-14868
Buckwheat	10	DOI: 10.1080/09064710.2015.1087587
Rice	10	https://doi.org/10.1016/j.stress.2023.100226

The resulting calculations Cumulative GDD was then categorized into 4 classes:

Low GDD: (0 - 500) - Insufficient for optimal growth
Moderate GDD: (501 - 1000) - Adequate for growth
High GDD: (1001 - 1500) - Ideal for development
Very High GDD: (1501+) - Optimal but may indicate heat stress
